# Krakencoder: a unified brain connectome translation and fusion tool

**DOI:** 10.1038/s41592-025-02706-2

**Published:** 2025-06-05

**Authors:** Keith W. Jamison, Zijin Gu, Qinxin Wang, Ceren Tozlu, Mert R. Sabuncu, Amy Kuceyeski

**Affiliations:** 1 Department of Computational Biology, Cornell University, Ithaca, NY USA; 2 Department of Radiology, Weill Cornell Medicine, New York, NY USA; 3 School of Electrical and Computer Engineering, Cornell University and Cornell Tech, New York, NY USA; 4 Department of Biomedical Engineering, Tsinghua University, Beijing, China

**Keywords:** Computational neuroscience, Neuroscience

## Abstract

Brain connectivity can be estimated in many ways, depending on modality and processing strategy. Here, we present the Krakencoder, a joint connectome mapping tool that simultaneously bidirectionally translates between structural and functional connectivity, and between different atlases and processing choices via a common latent representation. These mappings demonstrate exceptional accuracy and individual-level identifiability; the mapping between structural and functional connectivity has identifiability 42–54% higher than existing models. The Krakencoder combines all connectome flavors via a shared low-dimensional latent space. This fusion representation better reflects familial relatedness, preserves age- and sex-relevant information, and enhances cognition-relevant information. The Krakencoder can be applied, without retraining, to new out-of-distribution data while still preserving inter-individual differences in the connectome predictions and familial relationships in the latent representations. The Krakencoder is a notable leap forward in capturing the relationship between multimodal brain connectomes in an individualized, behaviorally and demographically relevant way.

## Abstract (web summary)

The Krakencoder can translate between structural and functional connectivity as well as between different atlases and processing choices via latent representations, as illustrated with datasets from the Human Connectome Project.

## Main

The brain is a vastly complex, interconnected network of neurons (and other types of cells), the healthy function of which enables us to move, think, feel, and observe, as well as interact with our environment. Studying how the brain’s connections relate to behavior, so-called brain connectome–behavior mapping, is crucial not only for understanding how the brain works generally but also for identifying biomarkers of disease, predicting outcomes in neurological disorders, and designing personalized interventions. Brain connectivity can be probed through various structural and functional neuroimaging techniques. Structural connectivity (SC) can be captured with diffusion magnetic resonance imaging (dMRI) measures of white matter, or the anatomical wiring, that connects different brain regions. Functional connectivity (FC), as measured with blood oxygen level-dependent functional MRI (BOLD-fMRI), quantifies the temporal similarity between brain regions’ dynamic neural activity patterns, irrespective of their structural connection. The association between SC and FC is a subject of intense research, given that it is believed that structural pathways provide the backbone over which functional activation flows. Despite this, correlations of SC and FC are moderate at best. Understanding the relationship between SC and FC is key to deciphering how the brain’s physical structure supports its dynamic functions and may offer insights into the neural mechanisms underlying behavior, injury or disease, and recovery^1–4^.

There are many approaches to modeling the relationship between SC and FC, most of which focus on using SC to predict FC. These methods include biophysical models (for example, linked neural mass models^5–10^), graph theoretical models (for example, communication strategies^11–15^), statistical models (that is, models of SC and FC topological metrics^16–19^) and network control theory^20–23^. More recently, machine learning has been used to predict FC from SC^24–27^, predict SC from FC^28^, or to translate a connectivity estimate between parcellations^29,30^. Many of these studies have shown that metrics of SC–FC relationships vary with age, sex and behavioral variables^16,27,31^, or disease and/or injury and recovery^4,32,33^.

Among the challenges and considerations in modeling this relationship, the assumption that estimated SC is an objective, fixed constraint on possible FC patterns is increasingly questioned. Evidence suggests that the brain’s functional dynamics can be highly flexible and adaptive, and may be driven by geometric factors not measured with SC^34^. Variability in SC estimation techniques, particularly in their sensitivity to detect faint or indirect connections, further complicates the interpretation of connectivity data. Moreover, the performance metrics and loss functions currently used in connectivity studies often fail to adequately account for inter-subject variability, potentially skewing the results and interpretations.

Despite the utility of modeling the relationship between connectomes, there is strong methodological disagreement in the literature as to how to extract the connectomes themselves from brain images^35–37^. Biases inherent in different processing choices, referred to here as ‘flavors’, have been shown to lead to different or even contradictory conclusions^38,39^. Each connectome flavor may differ in how the brain is parcellated into regions, how potential noise, artifacts, or confounds are removed, and how the connectivity between regions is quantified, among other methodological choices. Furthermore, connectome data from different studies are often shared only in certain flavors, making comparisons between datasets challenging.

In this work, our fundamental assumption is that each set of choices in the imaging and processing pipelines provides a different view of the same underlying system. Drawing inspiration from recent advances in multi-view learning^40^, we develop and share a tool called ‘Krakencoder’ that provides a way to combine these choices (that is, connectome fusion), and thus create a more comprehensive representation of the brain’s connectivity. Deriving a unified latent representation from within-modality and/or across modality connectome estimates may enable reconciliation of differing views of the same underlying network to overcome limitations and combine benefits from various processing choices. Krakencoder is a ‘universal’ brain connectivity encoding architecture capable of transforming connectivity estimates between parcellations, estimation techniques and modalities with high precision. Importantly, the Krakencoder is able to preserve inter-individual differences in a way that is behaviorally and demographically relevant. This element is crucial if we are to use the Krakencoder to improve our understanding of how brain structure and function and their interplay maps to behavior, impairment, or recovery after disease or injury.

## Results

### Krakencoder model architecture and training

**Fig. 1:**
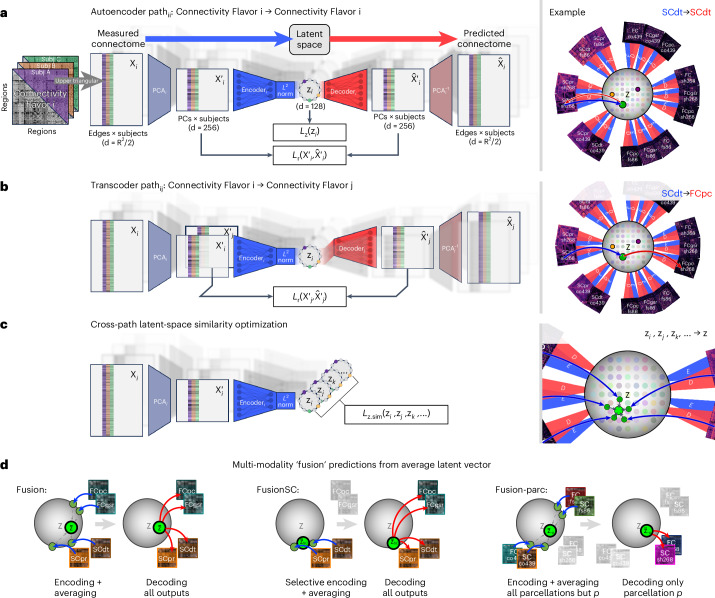
Key features of the Krakencoder architecture. **a**, Autoencoder path_*ii*_: connectivity flavor *i* to connectivity flavor *i*. This path begins by stacking the upper triangular portion of each subject’s connectivity matrix into input ${X}_{i}\in {{\mathbb{R}}}^{{n}_{{\rm{edges}}}\times {n}_{{\rm{subj}}}}$. A precomputed, fixed PCA transformation normalizes the data and reduces dimensionality to ${X}_{i}^{{\prime} }\in {{\mathbb{R}}}^{256\times {n}_{{\rm{subj}}}}$, equalizing the size of disparate input flavors. A single fully connected layer Encoder_*i*_, followed by *L*^2^ normalization, transforms ${X}_{i}^{{\prime} }$ into a latent hypersphere surface ${z}_{i}\in {{\mathbb{R}}}^{128\times {n}_{{\rm{subj}}}}$. Batch-wise encoding loss *L*_*z*_(*z*_*i*_) controls inter-subject separation in latent space. A single fully connected layer Decoder_*i*_ transforms *z*_*i*_ to ${\hat{X}}_{i}^{{\prime} }$, and batch-wise reconstruction loss ${L}_{r}({X}_{i}^{{\prime} },{\hat{X}}_{i}^{{\prime} })$ and *L*_*z*_(*z*_*i*_) are backpropagated to optimize Encoder_*i*_ and Decoder_*i*_. **b**, Transcoder *path*_*ij*_: connectivity flavor *i* to connectivity flavor *j*. This path converting input flavor *i* to output flavor *j* begins the same as path_*ii*_, transforming ${X}_{i}\to {X}_{i}^{{\prime} }\to {z}_{i}$, then Decoder_*j*_ transforms ${z}_{i}\to {\hat{X}}_{j}^{{\prime} }$, and reconstruction loss ${L}_{r}({X}_{j}^{{\prime} },{\hat{X}}_{j}^{{\prime} })$ is backpropagated to optimize Encoder_*i*_ and *Decoder*_*j*_. **c**, Cross-path latent-space similarity optimization. The latent similarity loss *L*_*z*.*sim*_(*z*_*i*_, *z*_*j*_, *z*_*k*_, …) provides explicit control to ensure that the latent representations of each subject are consistent across connectivity flavors. See Extended Data Table 1 for details about the loss terms. **d**, Multimodality fusion predictions from averaged latent space vectors. For these predictions we average the encoded latent vector from all input flavors (as in the fusion model), or a subset of input flavors (as in the fusionSC model, which averages latent vectors from only the SC inputs), and then decode this average vector to all output flavors. The fusion-parc model demonstrates cross-parcellation prediction, by averaging inputs from only the other parcellations.

The Krakencoder architecture consists of a set of encoders and decoders that enable transformation of each connectivity type into every other connectivity type via a common low-dimensional latent representation (Fig. 1). The Krakencoder was trained to optimize for both reconstruction accuracy and preservation of individual differences. The latter was achieved by enforcing latent-space representations of different connectivity types from the same subject to be close to one another and far from other subjects’ latent space connectome representations. The model was trained on resting-state FC and white-matter SC data from 683 healthy young adults (366 female, aged 28.6 ± 3.8 years) from the Human Connectome Project, validated during training on 79 held-out subjects (43 female, aged 29.1 ± 3.8 years), and finally tested on 196 subjects (107 female, aged 29.1 ± 3.6 years). Care was taken to ensure that no siblings spanned the divisions, because this data leakage can lead to over-optimistic model performance. Connectivity flavors consist of three parcellations (with 86, 268 and 439 regions), three common FC estimates (Pearson correlation, Pearson correlation following global signal regression, and regularized partial correlation), and two SC estimates (volume-normalized streamline counts from deterministic and probabilistic tractography), for a total of 15.

**Extended Data Fig. 1:**
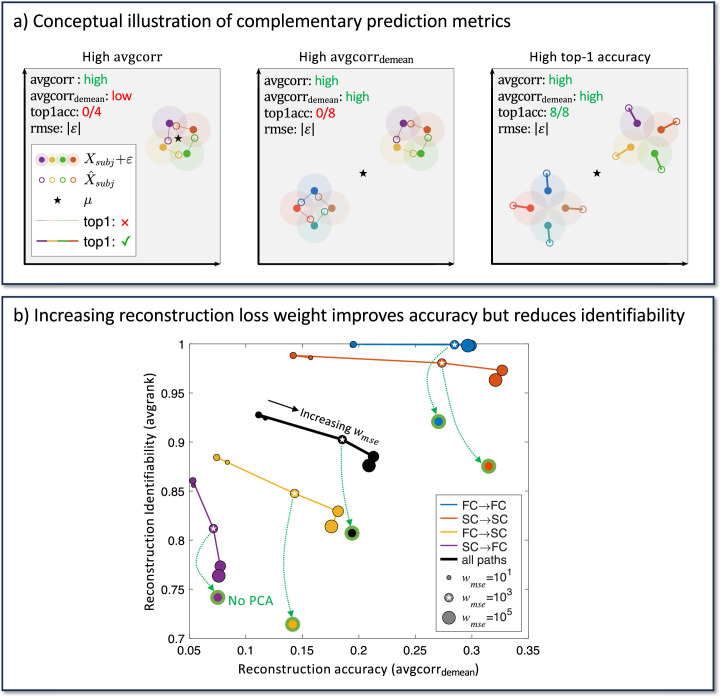
Connectome prediction similarity and identifiability are complementary. **a**. In this illustration, filled circles represent each subject’s measured connectome (*X*_*subj*_), open circles represent each subject’s predicted connectome (${\hat{X}}_{subj}$), and the black star is the population mean of all *X*_*subj*_. Thick lines indicate that a subject’s *X*_*s*=*a*_ is closer to its own ${\hat{X}}_{s = a}$ than any other ${\hat{X}}_{s\ne a}$. All three scenarios have the same residual magnitude ∣*ε*∣ (that is, RMSE), and similar average predicted correlation, but very different measured → predicted identifiability: Predictions for a given subject (${\hat{X}}_{subj}$) can be closer to the measured data of another subject than their own measurement (*X*_*subj*_). This relationship becomes complementary when observed data are close to the population mean or have distinct clusters. **b**. Each point shows the performance (reconstruction accuracy avgcorr_demean_, and identifiability *avgrank*) averaged within prediction categories for a model trained with a different reconstruction loss weight *w*_*mse*_. Intra-modality predictions (SC → SC in red, FC → FC in blue) have high identifiability, and increasing *w*_*mse*_ increases reconstruction accuracy without decreasing identifiability. Inter-modality predictions (FC → SC in yellow, SC → FC in purple), have lower accuracy and identifiability, and SC → FC identifiability decreases sharply with increasing *w*_*mse*_. *w*_*mse*_= 1000 was chosen to balance these two factors. Performance of a model trained without using PCA dimensionality reduction (green outlines) shows reduced ‘avgrank’ without improving avgcorr_demean_.

We create a separate encoder and decoder for each of the 15 connectivity flavors and then train the model to predict each flavor from each other flavor. There are 225 total training paths, including 15 autoencoders that map between the same flavor (Fig. 1a) and 210 transcoders that predict one flavor from another (Fig. 1b). Each encoder is a single fully connected linear layer, followed by *L*^2^ normalization that constrains the latent representation to the surface of a 128-dimensional hypersphere. Each decoder is a single fully connected linear layer. Dimensionality of the connectivity flavors varies between parcellations, which could lead to an imbalance in the training to favor higher dimension connectivity flavors. We balanced the contributions of each flavor by reducing each connectivity input to 256 dimensions using a precomputed principal component analysis (PCA), derived using only training data to avoid leakage between train and test sets. Reconstruction loss for each training path includes both Pearson correlation and Euclidean distance (Extended Data Table 1). We include a contrastive component to the correlation and Euclidean loss functions, to explicitly preserve inter-subject variability of predicted connectomes. A contrastive loss term is applied to the latent representation as well, to further promote inter-subject variability of the representations. The term ‘contrastive’ here refers to a penalty that attempts to force the same subject’s reconstructed and latent space connectomes close to each other and far apart from other individuals’ representations. Finally, we include an intra-subject latent space consistency loss to maximize the similarity of the latent representations from each connectome flavor for a given subject (Fig. 1c). This combination of loss functions enabled us to fine-tune the trade-off between reconstruction accuracy and inter-subject variability (Extended Data Fig. 1b), and to condition the latent space representation.

During model inference, the Krakencoder predicts each of the 15 connectivity flavors from any other, transforming predictions that exist in the reduced 256-dimensional space to their higher native dimensions using the inverse of the precomputed PCA transforms. The model can also combine information from multiple connectomes by averaging their latent space representations to obtain a ‘fusion’ representation; this fusion representation can then be decoded to predict all 15 connectome flavors. The encoding and averaging step in the fusion process can include all available connectome flavors, or a subset of flavors to, for example, predict FC flavors from the fusion latent representation of all SC flavors or vice versa (‘fusionSC’ or ‘fusionFC’, Fig. 1d), or to predict one parcellation using inputs from the averaged latent representations of all other parcellations (‘fusion-parc’, Fig. 1d). The fusion-parc version is particularly informative if one is using the Krakencoder to translate connectome data to a desired atlas on which it had not been computed.

### Connectome prediction performance

Given the high degree of similarity of measured connectomes to each other and to the population mean, we assess our connectome predictions using complementary measures of prediction identifiability (top-1 accuracy and average rank percentile, or avgrank, equation (4)) and de-meaned correlation (Pearson correlation after subtracting the population mean, or avgcorr_demean_, equation (2)). The accuracy of the Krakencoder’s connectome predictions was evaluated on 196 held-out test subjects.

**Fig. 2:**
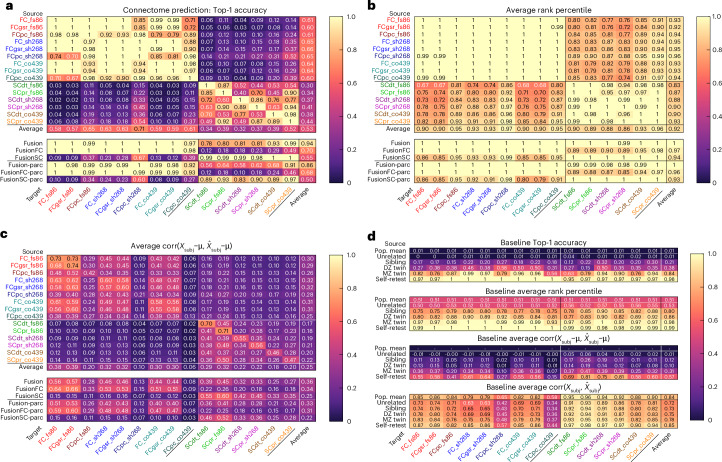
Connectome prediction performance for all input and output flavors on 196 held-out HCP-YA subjects. Heatmaps in **a**–**c** show connectome prediction performance from each source flavor (row) to each target flavor (column). Flavors consist of three parcellations (the 86-region FreeSurfer atlas (FS86), the 268-region Shen atlas (sh268) and the 439-region CocoHCP439 atlas (coco439 or co439)), three FC estimates (Pearson (FC), global signal regressed Pearson (FCgsr) and regularized partial correlation (FCpc)), and two SC estimates (deterministic tractography (SCdt) and probabilistic tractography (SCpr)). The Average row is the mean of performance metrics for all sources in the column above. The Fusion row shows predictions from the average latent vector from all 15 connectome flavors (Fig. 1d). FusionFC and FusionSC are predicted from the average of nine FC or six SC latent vectors, respectively. Fusion-parc predicts each parcellation from the average of all parcellations except itself. **a**, Connectome prediction: top-1 accuracy. This measures the fraction of test subjects whose predicted connectome is closer to their own measured connectome than to any other subject’s measured connectome. Chance level is 1/196 = 0.005. **b**, Average rank percentile. This measures the fraction of other subjects’ measured connectomes that are further from the target subject’s predicted connectome than the target subject’s own measured connectome. This rank percentile is then averaged across subjects. The chance level is 0.5. **c**, Average corr($\hat{\rm{X}}$_subj_ *−* μ*,* $\hat{\rm{X}}$_subj_ − μ). This is the average correlation of each subject’s measured and predicted connectomes, after subtracting the population mean (avgcorr_demean_). **d**, Baseline using related subjects as prediction. For each subject *s*_*a*_, we select an age- and sex-matched individual *s*_*b*_ of a given relatedness (unrelated, sibling, dizygotic (DZ) or monozygotic (MZ) twin, or self-retest) and consider the measured connectome from *s*_*b*_ as the prediction for *s*_*a*_. For each relatedness level, we compare the set of pseudo-predictions to measured connectomes using each performance metric, as though they were predictions from our model. Thus, we can observe the reliability and utility of each flavor and performance metric, and provide multiple baselines for evaluating model performance. We also show performance using the population mean for each flavor (‘pop. mean’) as pseudo-prediction, which for the avgcorr metric (bottom) exceeds self-retest, but for all other metrics is zero or chance.

The average rank percentile for FCs predicted from FCs of different parcellations or connectivity estimations (FC → FC) shows near-perfect identifiability (Fig. 2b, upper section, upper left block, mean = 1.0). Similarly, SC → SC predictions have near-perfect identifiability (upper section, lower right block, mean = 0.99). Inter-modality predictions performed worse than within-modality, but still far exceeding chance (random chance for avgrank is 0.5), with an overall average rank identifiability of 0.82 for SC → FC (lower left block) and 0.85 for FC → SC (upper right block). In general, we find that better avgrank prediction identifiability coincides with connectome sparsity, with higher dimensional parcellations (for example, the Coco439 atlas) and more sparse FC estimates (FC_pcorr_) being more easily identifiable. The avgcorr_demean_ metric (Fig. 2c) shows an average of 0.38 for FC → FC, and an average of 0.35 for SC → SC. Inter-modality predictions are again more difficult, with an overall avgcorr_demean_ of 0.16 for FC → SC of 0.16 and 0.09 for SC → FC. Lower dimensional parcellations generally had higher avgcorr_demean_. Higher dimensional atlases are likely to be better at capturing inter-individual variability in the functional boundaries within an atlas and thus have higher identifiability, while lower dimensional atlases are likely to benefit from higher signal-to-noise ratio in the measured regional SC and FC and thus have smaller reconstruction error when mapping between flavors.

Predictions of regularized partial correlation FC (FC_pcorr_) had the highest avgrank, but the lowest avgcorr_demean_ among FC flavors, which reflects the noise inherent in this estimate, even with regularization, but also an increased sparsity that drives identifiability. SC predicted from FC_pcorr_ yielded consistently better performance than other FC inputs, likely to be due to the increased sparsity and removal of nonspecific correlations. The best single FC input for predicting all SC flavors was the Shen268 FC_pcorr_, which predicts Coco439 SC_pr_ (probabilistic SC) with an avgrank of 0.99 and avgcorr_demean_ of 0.19. The best single SC input for predicting all FC flavors was the Coco439 SC_pr_. The fusion-parc predictions exclude inputs of the same parcellation from the averaged latent vectors, and their relatively high values demonstrate the level of shared information across flavors within the Krakencoder’s unified latent space.

**Extended Data Fig. 2:**
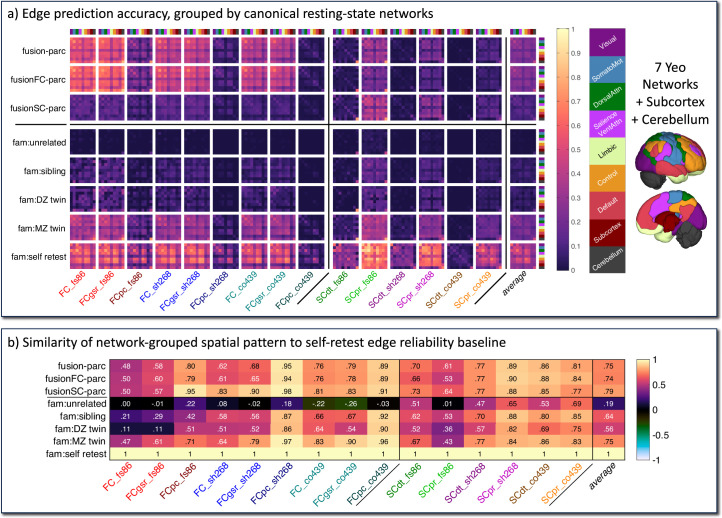
Spatial variation in edge prediction accuracy and baseline reliability. For the Krakencoder’s fusion predictions made without the parcellation being predicted (fusion-parc, fusionFC-parc, and fusionSC-parc), the prediction accuracy for each edge is the Pearson correlation between the predicted and measured values for 196 held-out test subjects. **a**. To simplify visualization and compare across parcellations, each region is assigned to one of 7 cortical resting-state networks^73^ or separate subcortical and cerebellar networks, and the edge-wise accuracies (region × region) are then grouped and averaged into a 9 × 9 network representation. Age- and sex-matched individuals of given relatedness, as well as self-retest, are correlated and averaged in the same manner, in order to provide a baseline for comparing the spatial patterns of edge-wise accuracy. **b**. Each network × network heatmap in **a** is correlated with the corresponding self-retest (bottom row), to show the consistency of the spatial pattern of edge-wise predictions with measurement reliability.

**Extended Data Fig. 3:**
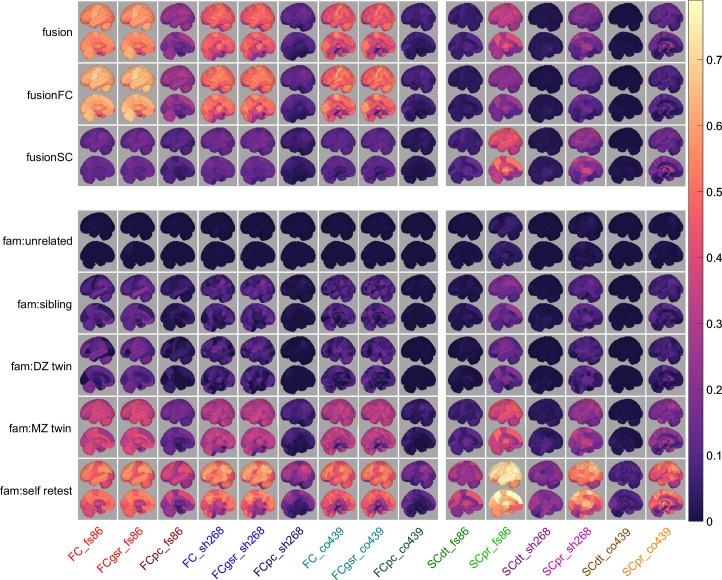
Regional variation in prediction accuracy and baseline reliability. For the Krakencoder’s fusion predictions made without the parcellation being predicted (fusion-parc, fusionFC-parc, and fusionSC-parc), the prediction accuracy for each region is the Pearson correlation between the predicted and measured edge values for 196 held-out test subjects, averaged across all other regions. Age- and sex-matched individuals of given relatedness, as well as self-retest, are correlated and averaged in the same manner, in order to provide a baseline for comparing the spatial patterns of edge-wise accuracy. Only the left hemisphere is shown, to simplify visualization.

**Extended Data Fig. 4:**
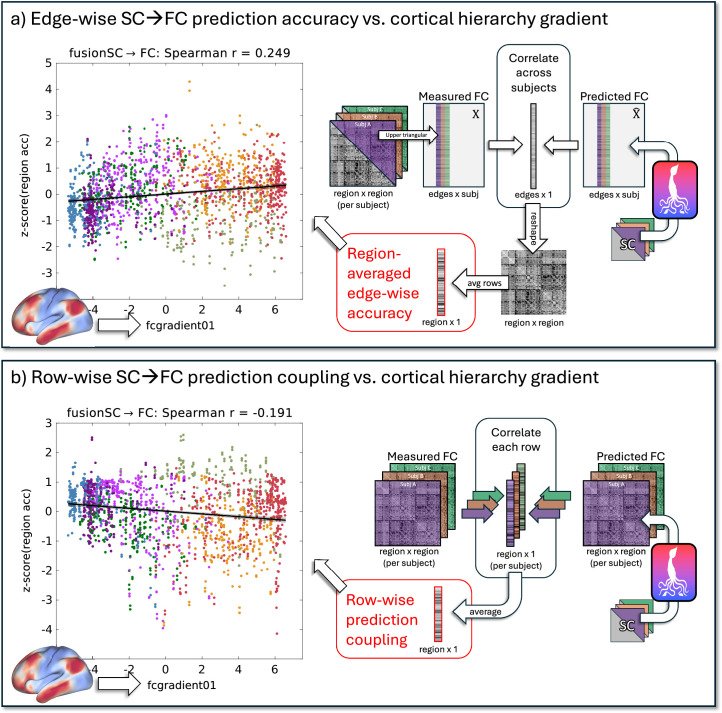
Inter-modality prediction accuracy aligns with cortical hierarchy. Regional SC → FC prediction accuracy covaries with the principal gradient of cortical hierarchy derived from functional connectivity^74^. **a**. Edge-wise prediction accuracy, measuring the explained inter-subject variance for each connectome edge, compared to fcgradient01. **b**. Regional prediction coupling, computed by correlating each region’s row in the measured and predicted connectome, compared to fcgradient01. Red (fcgradient01 > 0) and blue (fcgradient01 < 0) areas on the cortical map correspond to high-level association cortex and low-level sensorimotor areas, respectively. Each dot in the scatter plots represents a region colored according to its assigned Yeo network^73^.

In assessing the spatial variability of prediction accuracy, we find that regions with higher measurement reliability (for example, default mode) also have higher prediction accuracy (Extended Data Figs. 2 and 3). Furthermore, high-level association cortex has higher prediction accuracy than low-level sensorimotor cortex (Extended Data Fig. 4a, Spearman *r* = 0.249, *P*_spin_ = 0.019, 10,000 spin-test rotations). Conversely, as shown in previous work, regional structure–function coupling is lower for higher level cortex^16,19^ (Extended Data Fig. 4b, Spearman *r* = −0.191, *P*_spin_ = 0.116). This latter relationship primarily reflects the relationship between population-average SC and FC rather than predicting inter-individual variability.

**Extended Data Fig. 5:**
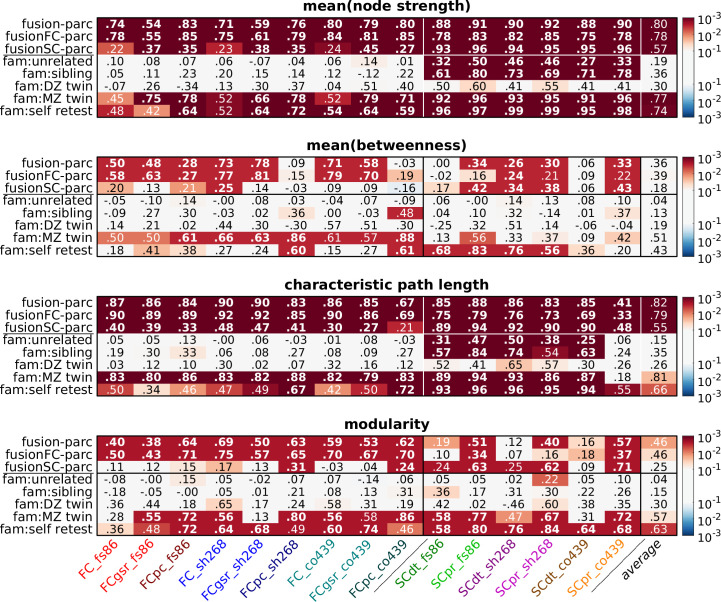
The Krakencoder preserves network properties of the connectomes. Each numbered entry in the heatmap shows the Spearman rank correlation between the graph metric of the Krakencoder’s predicted connectome (based on one of the three fusion representations, ‘fusion-parc’, ‘fusionFC-parc’, and ‘fusionSC-parc’, where inputs exclude the parcellation being predicted) for that output connectome flavor listed on the x-axis, compared to the graph metric for that measured flavor of connectome. These values were calculated using the held-out set of 196 individuals. Colors indicate the correlation’s p-value. Bold entries denote significant correlation (*p*_*perm*_ < 10^−3^, two-sided permutation test, 1000 permutations, FDR-corrected across 120 comparisons per metric).

Finally, we show that the Krakencoder’s fusion prediction also preserves the network properties of the measured connectomes. Extended Data Fig. 5 shows the Spearman correlation between the measured connectomes’ network properties (mean node strength, mean betweenness centrality, characteristic path length and modularity) and the Krakencoder’s predicted connectomes’ network properties, across 196 held-out individuals in the test set. The three fusion representations are used as input and each of the 15 connectome flavors as output; correlations between measured network metrics from the various familial relatedness groups are shown as comparison. The Krakencoder’s predicted connectomes generally preserve the network properties of the measured connectomes as well as or better than the correlation between network properties extracted from identical twins (that is, monozygotic) or two scans of the same individual (self-retest).

### Connectome similarity of related individuals

The fusionSC-parc and fusionFC-parc rows in Fig. 2a–c represent the accuracy of the Krakencoder when predicting an SC or FC connectome flavor from all other SC or FC flavors (excluding those from the predicted parcellation). If we compare this directly with Fig. 2d, we can see how the Krakencoder’s predicted connectome accuracy compares with several baseline estimates: measured connectomes from age- and sex-matched individuals who are unrelated, family members (non-twin siblings, dizygotic (DZ) and monozygotic (MZ) twins), retest sessions from the same individual (self-retest), or the population mean connectome. For instance, the self-retest row in the Fig. 2d heatmaps can be considered as a measurement reliability ceiling, while the ‘unrelated’ row shows the performance that can be explained by age- and sex-matching alone. Within a modality, top-1 accuracies (ranging from 0.93 to 1 for fusionFC-parc → FC and 0.83–0.97 for fusionSC-parc → SC) and average rank percentiles (all equal to 1 for fusionFC-parc and fusionSC-parc) in Fig. 2a and 2b are similar to the self-retest identifiability (when connectomes derived from imaging the same person twice are used to perform the identification), and better than the identifiability of individuals who are genetically identical (MZ twins). Similarly, the diagonal entries in Fig. 2c, representing the autoencoder avgcorr_demean_ for each flavor, range from 0.13 to 0.74, which is comparable to the self-retest noise ceiling (0.15–0.81), and are larger than the ‘MZ twin’ row (0.07–0.47, a 40–110% increase). This indicates that the autoencoders with a 128-dimensional bottleneck preserve more inter-subject connectome variability than can be explained by identical genetics, and approximately the same amount of variability as expected when measuring the connectome from the same individual twice. The fusionSC and fusionFC rows in Fig. 2c represent the avgcorr_demean_ for the unified SC (or FC) → FC (or SC) connectome predictions, with values ranging from 0.03 to 0.16 for SC → FC and 0.16–0.26 depending on the flavor. These exceed the similarity observed in the sibling or ‘DZ twin’ row from the avgcorr_demean_ subpanel in Fig. 2d, indicating that predicting FC from our unified SC representation preserves more inter-subject FC variability than can be explained by age, sex and 50% genetic similarity.

### Demographic and behavioral prediction from latent space

**Fig. 3:**
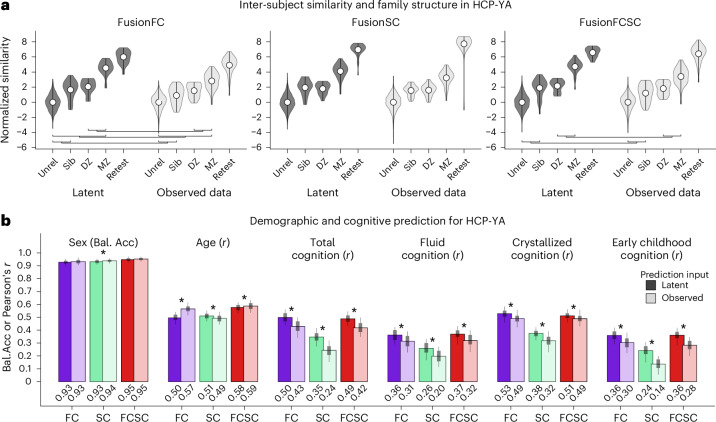
Familial connectome similarity and demographic prediction for held-out HCP-YA subjects. **a**, Inter-subject similarity of latent space representations and observed SC and FC data, stratified by family relationship. Each violin plot shows the distribution of inter-subject similarity for age- and sex-matched pairs for each familial relationship (*n* = 60 sibling pairs, 26 DZ, 58 MZ), as well as unrelated pairs (*n* = 8,694) and self-retest (*n* = 37). Due to the high baseline similarity in observed data, latent and observed data similarity measures were independently z-scored by the mean and standard deviation of the similarity of unrelated subjects for visual comparison. Separability of each pair of violins within a modality was computed as the area under the ROC curve. Separability estimates for a given relationship pair from the latent space and observed data were statistically compared using a permutation test, where bars show family-level pairs where latent space separability exceeds observed data separability (*P*_perm_ = 0 to 0.015, two-sided permutation test, 10,000 permutations, FDR-corrected across 30 comparisons). **b**, Prediction accuracy of demographic and cognitive metrics from different subsets of the Krakencoder’s latent space and observed SC and FC data. Variability of prediction accuracy was assessed through bootstrap resampling (*n* = 100). Bar height, box and whiskers represent the mean, 50% and 90% bootstrap confidence intervals, respectively. *Significant difference between prediction accuracy from latent space and the observed data (*P*_perm_ < 10^−4^, two-sided permutation test, 10,000 permutations, FDR-corrected across 18 comparisons). Bal.Acc, balanced accuracy.

We next tested whether the Krakencoder latent space preserves inter-subject variation related to family structure as well as demographic and behavioral features. We examined the inter-subject similarity of latent space representations for pairs of age- and sex-matched unrelated individuals, non-twin siblings, DZ and MZ twins, and self-retest, along with inter-subject similarity of observed connectomes (Fig. 3a). By comparing the receiver operating characteristic (ROC) separability of each pair of violin plots, we see that separation is greater for the Krakencoder latent space than for the observed connectomes, with statistical significance for unrelated versus sibling, DZ versus MZ twins, and MZ versus unrelated (*P*_perm_ = 0 to 0.015, two-sided permutation test with 10,000 permutations, false discovery rate (FDR)-corrected across all 30 comparisons).

To demonstrate the preservation of demographically and behaviorally relevant features in the Krakencoder latent space, we trained linear models to predict individuals’ age, sex and cognitive scores (‘total’, ‘fluid’, ‘crystallized’ and ‘early childhood’ composite scores from the NIH (National Institutes of Health) toolbox) from the Krakencoder latent space, and compared them with the same predictions based on observed connectome data. Linear kernel ridge regression models for each variable were built from the mean latent vectors (fusionFC, fusionSC, fusionFCSC, each of which is *n*_subj_ × 128) or from the average inter-subject cosine similarity of observed data (that is, the average of 9 FC, 6 SC or 15 SC + FC *n*_subj_ × *n*_subj_ matrices) (Fig. 3b). The Krakencoder and observed connectome data predict sex with a similar balanced accuracy of ≥0.93. The Krakencoder latent space predicts age with Pearson *r* ≥ 0.5 (fusionFC *r* = 0.50, mean absolute error (MAE) = 2.35; fusionSC *r* = 0.51, MAE = 2.57; fusionFCSC *r* = 0.58, MAE = 2.33), similar to the predictions from observed data (observed FC *r* = 0.57, MAE = 2.31; observed SC *r* = 0.49, MAE = 2.58; observed FC + SC *r* = 0.59, MAE = 2.33). For age prediction, fusionSC outperformed observed SC, and observed FC outperformed fusionFC (*P*_perm_ < 10^−4^, two-sided, 10,000 permutations, FDR-corrected across all 18 comparisons), but the observed data and fusion SCFC models performed similarly. The Krakencoder latent space predictions significantly outperformed the observed data model predictions for predicting all cognitive composite scores (*P*_perm_ < 10^−4^, two-sided permutation test with 10,000 permutations, FDR-corrected across all 18 comparisons).

### Sensitivity analyses

**Fig. 4:**
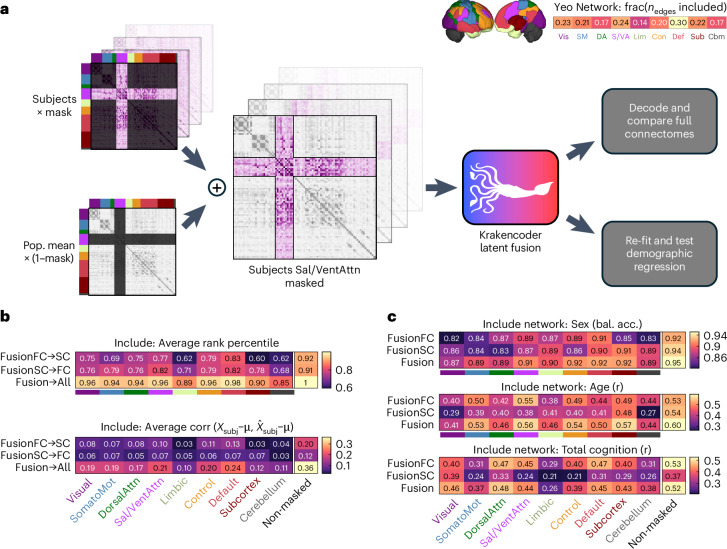
Spatial input sensitivity analysis of Krakencoder predictions. **a**, Brain network masking and analysis workflow. We estimate the sensitivity of the Krakencoder’s prediction accuracy to regions in a given Yeo functional network by replacing all connectome edge values not in that network with the population mean, and feeding these masked connectomes into the connectome and demographic predictions. The accuracy after masking reveals the amount of information that the particular network’s regional connections have in the mapping and the demographic and behavioral predictions. Upper right panel shows the overall number of edges in each network. bal.acc, balanced accuracy; Cbm, cerebellum; Con, control; DA, dorsal attention; Def, default; Lim, limbic; SM, somatomotor; Sub, subcortex; S/VA, salience/ventral attention; Vis, visual. **b**, Masked connectome predictions. Connectome prediction identifiability (avgrank, top) and reconstruction accuracy (avgcorr_demean_, bottom) using only the connections to and from regions within each brain network shows how much information the model is utilizing from each network when predicting whole-brain connectomes. The Non-masked column on the right shows performance using original connectomes without masking. Values that are lower indicate that the network’s connections do not contribute as much to the accuracy of the Krakencoder’s mapping. **c**, Similarly, predicting subject sex (top), age (middle), and total cognition (bottom) from masked input data shows the relative amount of information that the connections to and from regions in each network contain about those demographic and cognitive features.

Sensitivity analyses were performed to determine which brain areas’ connections played the largest role in the accuracy of the Krakencoder’s connectome mapper, and in the accuracy of the models predicting age, sex or total cognition from the Krakencoder latent space (Fig. 4a). These analyses demonstrate how the Krakencoder may be used to understand the biological underpinnings of, for example, SC to FC relationships, or which brain networks may play the largest role in predicting cognition.

By examining the mean avgrank and avgcorr_demean_ when retaining only the individual-level values of those networks’ connections, we see that default mode network connections appear important for mapping accuracy, particularly for the fusion → all and fusionFC → SC (Fig. 4b). Connections to and from regions in the somatomotor network and subcortex seem to be more informative for fusionSC → FC than for fusionFC → SC. Together, these heatmaps illustrate that although connections to a single network contain much of the information needed to identify an individual’s connectome (for example, avgrank from default mode network inputs is 98% of that from the non-masked input), it is not sufficient to numerically recreate all of the missing connections (for example, avgcorr_demean_ from default mode network retains 66% of the non-masked input). This may arise from a combination of factors including the difficulty of predicting so many missing features, and the measurement noise of those predicted features.

In Fig. 4c we see the largest retention in accuracy for predicting sex when functional and structural connections to and from regions in the default mode network and structural connections to and from the subcortex are retained. Age was best predicted using functional connections to and from the salience and ventral attention network, and structural connections to and from the subcortex. Total cognition was best predicted by functional connections to and from regions in default mode and dorsal attention networks, and by structural connections to and from regions in the visual network.

### Performance on out-of-sample, out-of-distribution data

**Fig. 5:**
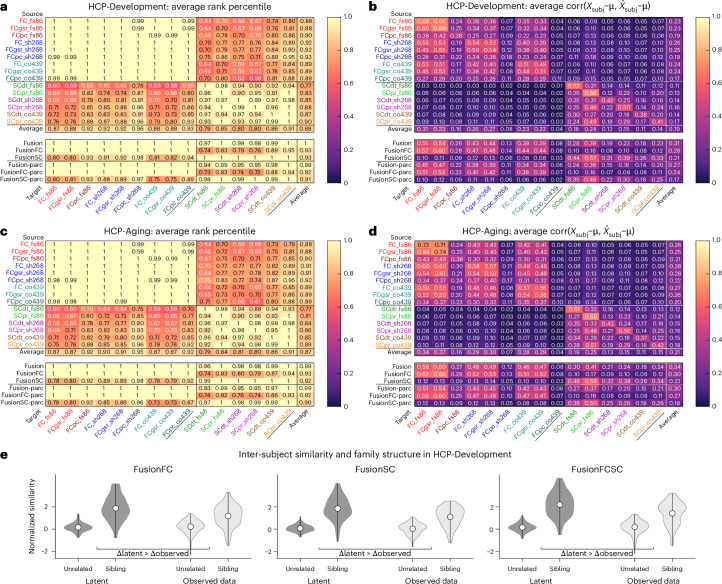
Out-of-sample performance of the Krakencoder on individuals from across the lifespan. **a**–**d**, Heatmaps of connectome prediction performance for subjects from the HCP-Development study (ages 8–21 years) (**a**,**b**) and the HCP-Aging study (ages 36–100 years) (**c**,**d**). The Krakencoder model applied was trained on HCP-YA data (ages 22–37 years) and frozen; no retraining or fine-tuning was performed. **a**,**c**, Average rank percentile (Fig. 2b). This is the fractional rank of the similarity (Pearson correlation) of a subject’s predicted and measured connectomes compared with all other measured connectomes, averaged across subjects (random chance, 0.5). **b**,**d**, Average corr ($\hat{\rm{X}}$_subj_ *−* μ*,* $\hat{\rm{X}}$_subj_ *−* μ). Average correlation of each subject’s measured and predicted connectomes, after subtracting the population mean. **e**, Sibling similarity from the HCP-Development study. Connectomes from 76 sibling pairs in HCP-D are more similar than age- and sex-matched unrelated pairs, with the latent space similarity of the sibling pairs being significantly more separable from that of the unrelated pairs than in the observed data. Each violin plot shows the distribution of inter-subject similarity for sibling pairs or pairs of unrelated individuals with matching age and sex disparity. As in Fig. 3a, latent and observed connectome similarity were independently normalized for visualization. Separability was measured using ROC analysis, and significance was assessed using permutation testing. For all three fusion types (fusionFC, fusionSC, fusionFCSC), siblings were more similar in the Krakencoder’s latent space than in the observed data (*P*_perm_ < 10^−4^, two-sided permutation test, 10,000 permutations, FDR-corrected across three comparisons).

To assess generalizability of the Krakencoder to new datasets, we applied our frozen, pretrained Krakencoder model to data from the multi-site Human Connectome Project (HCP) Lifespan study^41–43^, including both the Development cohort (HCP-D) and the Aging cohort (HCP-A). These two studies differ from the original HCP young adult (YA) study in both subject demographics (ages 8–21 years for HCP-D and 36–100+ years for HCP-A versus 22–37 years for HCP-YA) and acquisition parameters, with voxel sizes and scan durations more similar to those used in typical neuroimaging studies. To account for domain shift, we linearly mapped the population mean of each connectome flavor to the population mean of the HCP-YA training subjects before applying the HCP-YA-derived PCA dimensionality reduction and Krakencoder latent space projection. Despite the demographic and acquisition differences, the Krakencoder still predicts connectome flavors with high accuracy and precision (Fig. 5a–d). Within-modality connectome prediction identifiability remained high for both HCP-D and HCP-A (avgrank > 0.96). Inter-modal prediction identifiability was reduced 6–12% compared with held-out HCP-YA subjects, although with avgrank > 0.75 it was still well above chance. Intra-modal avgcorr_demean_ was reduced by 10–26%, while inter-modal avgcorr_demean_ was 30–50% lower than that for held-out HCP-YA. Thus, despite the reduction in prediction accuracy due to substantial differences in age and acquisition, the Krakencoder model still performs well above chance and preserves inter-individual differences when mapping between connectome flavors.

**Extended Data Fig. 6:**
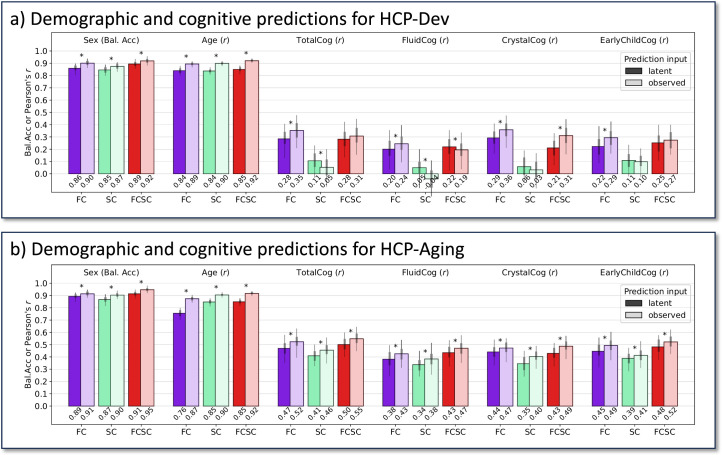
Demographic prediction for HCP-Lifespan (out-of-sample test set). Prediction accuracy of demographic and cognitive metrics from different subsets of the latent space and observed connectome data, for HCP-Dev (**a**) and HCP-Aging (**b**). Bar height, box, and whiskers represent the mean, 50%, and 90% bootstrap confidence intervals, respectively. * = significant difference between prediction accuracy from latent space and observed data. (*p*_*perm*_ < 10^−4^, two-sided permutation test, 10000 permutations, FDR-corrected across 18 comparisons).

The HCP-D dataset contains 76 sibling pairs (50 same-sex, 26 opposite sex, *Δ*age 0–9 years), therefore we performed a familial similarity analysis similar to that for HCP-YA. In Fig. 5e we show that siblings were more separable from age- and sex-matched individuals in latent space than in the observed data for all three fusion types (fusionFC, fusionSC, fusionFCSC) (*P*_perm_ < 10^−4^, two-sided permutation test, 10,000 permutations, FDR-corrected across 3 comparisons). Finally, we performed a regression analysis to predict age, sex and cognition in the HCP-D and HCP-A datasets (Extended Data Fig. 6). New predictive models using the same approach as that for the HCP-YA predictions were fitted and cross-validated for each of the two new datasets. As with HCP-YA, sex was predicted very accurately by both latent and observed data models (balanced held-out test accuracy = 0.86–0.95). Age was predicted with much higher accuracy in HCP-D and HCP-A than in HCP-YA (HCP-D *r* = 0.84–0.95, MAE = 1.45–1.72; HCP-A *r* = 0.76–0.85, MAE = 6.13–6.52 on held-out test data), likely to be due to the much larger age range and larger age-related changes during development and aging. Cognitive score prediction performance for the HCP-A data was similar to or better than HCP-YA, perhaps due to larger age-related cognitive effects, but performance was considerably worse for the HCP-D dataset. This decrease in predictive ability was especially pronounced when predicting cognition from SC, in which accuracy (Pearson’s *r*) from latent and observed data models was not significantly greater than 0. For all other predictions, observed data models performed significantly better than those on the Krakencoder latent space (*P*_perm_ < 10^−4^, two-sided permutation test, 10,000 permutations, FDR-corrected across 18 comparisons), although the Krakencoder predictions remained well above chance.

We aim to further test Krakencoder’s biological plausibility by applying it to connectomic data from individuals with pathological changes in the brain’s structural and functional connections. We extracted SC and FC from 100 individuals with multiple sclerosis (age, 22–71 years, 45.5 ± 11.4 years; 66% female; disease duration 12.97 ± 8.07 years), an autoimmune disease that results in lesions primarily in the brain’s white matter^44^. This disease has also been associated with changes in SC and FC that are related to individuals’ disability levels^45,46^. The fMRI and dMRI acquisitions for these patients had substantially lower spatial, temporal and angular resolution than the HCP-YA or HCP-Lifespan data (Extended Data Table 2). We fed the observed, pathological SC and FC into the corresponding arms of the frozen Krakencoder to obtain a fusionSC and fusionFC, from which we predicted the various FC or SC flavors. We found that the average rank identifiability of the Krakencoder’s predicted FC (from the fusionSC) was up to 83%, which was higher than the identifiability of the observed SC to observed FC (calculated within the same atlas), which was at most 55% (Supplementary Fig. 1a,b). Mapping the fusionFC, calculated from fMRI in the patients with multiple sclerosis, to the various SC flavors using the Krakencoder had around the same identifiability (up to 69%) as the mapping between observed FC to SC (up to 67%). Higher prediction performance for SC → FC than FC → SC is consistent with our out-of-sample HCP-Lifespan results. Using the current model and a simplistic approach to domain adaptation, our multiple sclerosis FC data appear less well matched to the HCP-YA FC training data than multiple sclerosis SC, or either HCP-Lifespan dataset (Supplementary Fig. 1c). This mismatch, due in part to differences in fMRI and dMRI acquisition, may explain the increased difficulty in making predictions from these inputs. Overall, these results show that even though the Krakencoder was trained on young, healthy individuals, it can still represent with good fidelity the relationship between connectomes in individuals with pathology. Importantly, it may indeed better capture the mapping from SC to FC than the observed connectome data itself. This provides evidence of the biological plausibility of the Krakencoder’s architecture and hints at its possible clinical relevance in capturing low-dimensional representations of disease pathology.

### Comparison with existing techniques

Although the prediction of FC from SC is not the only application of our model, this route of prediction is among the most commonly studied problems in brain connectivity mapping. To compare our current Krakencoder model with this previous literature, we gathered a few recent studies that used different approaches to predict FC from SC. The first is a deep neural network proposed by Sarwar et. al^24^ (‘deepnet’) and the second is a graph neural network from Neudorf et. al^25^ (‘graphnet’).

**Extended Data Fig. 7:**
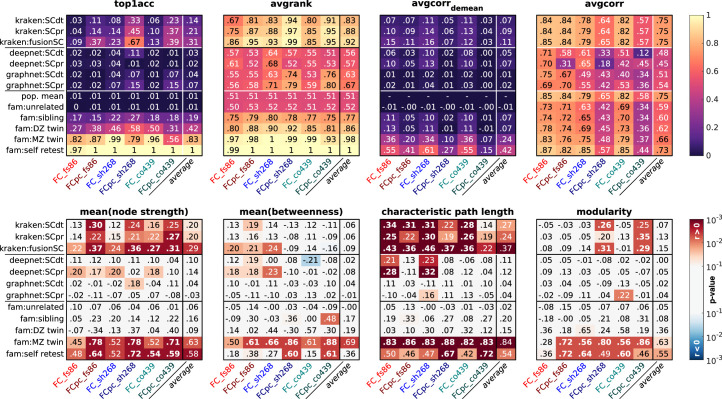
Comparison of the Krakencoder’s SC to FC mapping to state-of-the-art ML-based SC to FC mapping. Krakencoder’s performance in mapping from SC to FC, compared against a deep neural network (‘deepnet’)^24^ and a deep graph neural network (‘graphnet’)^25^. Top-1 accuracy (*top*1*acc*), average rank percentile (*avgrank*), average correlation of predicted and measured FC after (avgcorr_demean_) and before (*avgcorr*) de-meaning are presented in the top row of panels, one value for each FC flavor as output (columns) and various SCs as input (rows). Network metrics are provided in the bottom row of figure panels. There are three SC inputs for the Krakencoder model: volume-normalized deterministic SC for that parcellation only [kraken:SCdt], volume-normalized probabilistic SC for that parcellation only [kraken:SCpr] and the fusion of all 6 SC inputs in the latent space [kraken:fusionSC]. There are two SC input flavors (deterministic [SCdt] and probabilistic [SCpr] volume-normalized SC) for the deepnet and graphnet models. The same metrics between pairs of measured FC from varied familial relatedness categories is shown as a comparison. Deepnet and graphnet models were trained and evaluated using the same data and subject splits as the Krakencoder model. Colors indicate the correlation’s p-value. Bold entries denote significant correlation (*p*_*perm*_ < 10^−3^, two-sided permutation test, 1000 permutations, FDR-corrected across 72 comparisons per metric).

By both the avgrank and avgcorr_demean_ metrics, the predictions from our model outperform both the deepnet and graphnet models across all connectome flavors and parcellations (Extended Data Fig. 7). Specifically, the Krakencoder avgrank was 17–86% higher (average 42%) than the deepnet or graphnet models for the same single SC inputs, and 18–90% higher (average 54%) using the fusionSC input to predict each FC flavor. Krakencoder avgcorr_demean_ was 1–890% higher (average 335%) than either model using single SC inputs, and 25–980% higher (average 450%) using fusionSC. Similarly, the accuracy of network properties for FC predicted by Krakencoder exceeded that from either alternative SC to FC framework. These particular results highlight the special emphasis of the Krakencoder on preservation of individual differences when mapping from SC to FC.

### Extending the Krakencoder with new connectivity flavors

Given a new connectivity flavor computed on the same training subjects as the pretrained model, we can train a new autoencoder with an additional loss term to align the new latent representation with the precomputed fusion latent representation (Supplementary Fig. 2a). To illustrate this capability we extended our model to new data from a previously unseen parcellation (cc200, ref. ^47^), with bandpass-filtered time series for FC (original flavors use a high-pass filter) and un-normalized streamline count for SC (original flavors use pairwise volume normalization). Prediction identifiability and reconstruction accuracy are comparable to the original 15 flavors (Supplementary Fig. 2b). Unlike the training of the original 225-path model, which takes up to 30 hours on a GPU (graphics processing unit), the addition of each new flavor takes only 1–2 minutes without a GPU. In this example, we used the original 683 HCP-YA training subjects, but this model-extension technique can use any population for which we have already computed the Krakencoder latent representation.

## Discussion

Previous work in mapping between connectomes, mostly predicting FC from SC, has largely focused on optimizing accuracy in the model predictions by maximizing the correlation between predicted and measured FC. We argue here that over-emphasis on this metric can result in a model that predicts FC that is close to the population mean and/or does not preserve individual differences. The deepnet model did incorporate into its loss function a term that aimed to reduce inter-subject similarity of predicted FC^24^. However, it appears that this only pushed the individual FC predictions away from one another and not toward the particular individual’s measured connectomes, given that its identifiability was just above chance. The Krakencoder’s connectome predictions, however, achieve perfect average rank identifiability within the same connectome modality, and consistently high average rank identifiability when predicting FC from SC. Other modeling work has shown that regional SC–FC coupling follows a functional gradient wherein the coupling is highest in visual and somatomotor networks and lowest in higher-order association networks^16,19^.

The Krakencoder has many novel aspects, including its ability to translate between connectome flavors without requiring raw data. This is useful when researchers only have access to a connectome from a particular pipeline or atlas and need it in another. One previously developed tool called CAROT does enable translation of FC from one atlas to another without requiring the raw data^30^; however, that tool does not enable translation of connectomes between different processing pipelines or mapping between SC and FC. There is no specific FC or SC processing pipeline that has been agreed upon by the field; many researchers choose one pipeline and replicate on others. The ability of the Krakencoder to predict other flavors within the same modality suggests a common, latent connectomic backbone that gives rise to each FC or SC flavor when a given parcellation or connectome estimation strategy is used. The Krakencoder’s fusion functionality removes the burden of having to choose one pipeline or replicate findings on several different pipelines and attempt to make general conclusions. More than that, we believe that the fusion of various snapshots of the underlying connectome increases the signal-to-noise ratio of the connectome representations and perhaps becomes more than the sum of its parts. This latter statement is supported by the finding that models predicting cognition from the fused latent space representations outperformed models based on the observed connectome data; age and sex predictions were similar between the models but these are generally easier to predict compared with cognitive scores. As we have demonstrated, the Krakencoder’s signal-to-noise ratio boosting effect can be especially useful when the tool is applied to connectomes extracted from lower quality data, which is often the case in clinical populations.

The Krakencoder is robust when applied to completely new, out-of-distribution data from individuals across the lifespan (8–100+ years of age) and from adults with pathological changes to their connectomes (individuals with multiple sclerosis). The frozen model (trained on young adult data) still retained a high level of identifiability and explained variance when mapping between connectomes from the HCP-D, HCP-A and multiple sclerosis datasets. The Krakencoder appears somewhat impervious to the sweeping connectomic changes that occur throughout human development, aging and, to a certain extent, disease processes^48,49^. The inter-modality predictions become more difficult when new datasets are less consistent with the training data, with the current model more sensitive to domain mismatch in prediction inputs than outputs. The drop in demographic predictions of the latent space for the HCP-D, HCP-A and multiple sclerosis data could mean that the Krakencoder may benefit from further training (perhaps fine-tuning) using lifespan and/or disease data.

Future work will apply the Krakencoder to predict connectomes under other clinical conditions, and further explore the relationship between the common latent representation and factors of neuroscientific or clinical relevance. We will also create a fine-tuning procedure to further increase the robustness of the Krakencoder when applied to novel datasets, to promote its wider applicability, particularly in datasets that contain pathology such as stroke and Alzheimer’s disease. We have demonstrated the ability to train encoding and decoding arms for new connectome flavors, but the Krakencoder could also be adapted to data from other modalities (magneto- or electro-encephalography) or even to explicitly capture demographic and/or behavioral information so that the latent space may be used more effectively for brain–behavior mapping. While we purposefully chose atlases that were derived in varied ways, that is, using anatomical versus functional information, there still may be disagreement in the labeling of regions across subjects. However, by translating between and fusing SC and FC derived from all atlases, the Krakencoder attempts to identify a latent organizational representation that underlies those observed in different overlapping parcellation schemes. In the context of SC–FC coupling, we do not think that these atlas choices prevent its accurate evaluation within an individual, given that we are using the same parcellation for both SC and FC. Finally, while this first proof-of-concept work focused on applying the Krakencoder to static FC, we know that brain activity is flexible and adapts to task demands, varied states, and pharmacological manipulations. Further work will investigate the adaptation of the Krakencoder to better reflect dynamic changes that occur in brain networks.

## Methods

This research was approved by the Weill Cornell Medicine Institutional Review Board.

### Data description

The data for this study come from the original Human Connectome Project (HCP)^50^ and the HCP Lifespan studies^41–43^, which together include high-resolution 3 T MRI data for 2,490 healthy participants aged 8–100+ years. Primary model training and testing was performed using data from 958 subjects from the original young adult HCP study (HCP-YA), aged 22–37 years, which includes MZ and DZ twin pairs as well as non-twin siblings. For benchmarking performance, we include a subset of 40 HCP-YA subjects who returned for a complete retest session 1–11 months after their initial session. The HCP-Development (HCP-D, 608 subjects, aged 8–21 years, including 76 pairs of non-twin siblings) and HCP-Aging (HCP-A, 716 subjects aged 36–100 years) cohorts from HCP Lifespan were used to evaluate our pretrained model on out-of-sample, out-of-distribution data. HCP Lifespan data differ from HCP-YA in both age range and acquisition parameters, with Lifespan acquiring less than half as much fMRI and dMRI data as HCP-YA. HCP Lifespan studies were also collected across four different sites using standard MRI scanners while the HCP-YA data were all acquired on a custom MRI scanner at a single site. See Extended Data Table 2 for demographic and acquisition details. For all three datasets, we excluded subjects with incomplete resting-state fMRI or dMRI, and subjects with known acquisition or quality control issues.

### Construction of functional connectomes

All resting-state fMRI time series were preprocessed by HCP using the minimal preprocessing pipeline^51^, which includes motion correction, susceptibility distortion correction, coregistration to anatomy and nonlinear template space (FMRIB Software Library (FSL) MNI152 template, 6th generation), as well as automated denoising using ICA-FIX^52^. We used a custom post-processing pipeline to identify motion and global signal outlier timepoints (global signal > 5*σ*, motion derivative >0.9 mm), regress out tissue-specific nuisance time series (five eigenvectors each from eroded white matter and cerebrospinal fluid masks^53^) and motion-related time series (24 in total, including six motion parameters, their backward derivatives, and the squares of both^54^), and temporally filter (high-pass >0.01 Hz for HCP-YA, >0.008 Hz for HCP-A/D, using DCT (discrete cosine transform) projection), and parcellate time series using each atlas. Outlier timepoints were excluded from nuisance regression and temporal filtering. Regional time series for each brain region were obtained by averaging voxels in the denoised time series data. Regional time series for each of the four (for HCP-YA) or two (for HCP-Lifespan) scans were variance-normalized and concatenated, and Pearson correlation between regional time series (excluding outlier timepoints) resulted in a region × region resting-state FC matrix for each subject. We also computed an FC matrix with global signal regression, FC_gsr_, by regressing the mean gray matter time series and its temporal derivative from each regional time series before computing region × region Pearson correlation.

Finally, we computed a Tikhonov-regularized partial correlation FC_pcorr_ (or FCpc) that minimizes the average Euclidean norm between subject FC_pcorr_ and the population mean of the unregularized precision matrices^36^:

$$\begin{array}{rcl}\overline{\Omega }&=&\frac{1}{{n}_{{\mathrm{subj}}}}\mathop{\sum }\limits_{a}^{{n}_{{\mathrm{subj}}}}{\mathrm{F{C}}}_{a}^{-1}\quad \,\text{1. Compute population mean}\\&& \text{unregularized inverted}\,\,{\mathrm{FC}}\end{array}$$

$$\begin{array}{rcl}{\hat{\Omega }}_{a}&=&{[{\mathrm{F{C}}}_{a}+\lambda I]}^{-1}\quad \,\text{2. Find}\,\,\lambda \,\,\text{that minimizes mean squared}\\&& \text{error (MSE) loss between subject}\,\,\hat{\Omega }\,\,\text{and target}\,\,\overline{\Omega }\end{array}$$

$$\lambda ={{\rm{argmin}}}_{\lambda }\mathop{\sum }\limits_{a}^{{n}_{subj}}\parallel {\hat{\Omega }}_{a}-\overline{\Omega }{\parallel }_{2}$$

$$\begin{array}{rcl}{\mathrm{F{C}_{pcorr}}}(i,j)&=&-{\hat{\Omega }}_{ij}/\sqrt{{\hat{\Omega }}_{ii}{\hat{\Omega }}_{jj}}\quad \,\text{3. Normalize precision matrix}\\&&\hat{\Omega }\,\,\text{to compute partial correlation}\,\,{\mathrm{F{C}_{pcorr}}}\end{array}$$

For the Shen268 and Coco439 atlases, the target $\overline{\Omega }$ was computed by averaging the pseudoinverse of each subject FC rather than the inverse. A different *λ* was identified for each atlas, based on the 700 training subjects from HCP-YA. Optimal *λ* was 0.06 for FS86, 0.15 for Shen268 and 0.25 for Coco439. For the HCP-A, HCP-D and multiple sclerosis data, regularization target $\overline{\Omega }$ was the $\overline{\Omega }$ from the HCP-YA training data. For HCP-A: FS86 *λ* = 0.10, Shen268 *λ* = 0.31, Coco439 *λ* = 0.57. For HCP-D: FS86 *λ* = 0.17, Shen268 *λ* = 0.35, Coco439 *λ* = 0.54.

### Construction of structural connectomes

The dMRI data were preprocessed by HCP using the minimal preprocessing pipeline, which jointly corrected for motion, susceptibility distortion and eddy current distortion using FSL’s ‘topup’ and ‘eddy’ tools^55,56^, before being linearly coregistered to the anatomical image. Preprocessed dMRI were further processed using MRtrix3 (ref. ^57^; 3.0_RC3), including bias correction, constrained spherical deconvolution (multi-shell, multi-tissue fiber orientation distribution (FOD) estimation, lmax = 8, ref. ^58^), and whole-brain tractography. We performed both deterministic (SD_STREAM^59^, or SCdt) and probabilistic, anatomically constrained tractography (iFOD2+ACT^60,61^, or SCpr) using dynamic white-matter seeding^62^ for both methods, resulting in 5 million total streamlines per subject for each tractography algorithm. SC matrices were constructed for each atlas by counting the number of streamlines that ended in each pair of gray matter regions, normalized by the total volume of each region pair.

### Parcellations

We used three different whole-brain atlases, covering a range in size and construction methodology. The 86-region FreeSurfer atlas (FS86) combines 68 cortical gyri from Desikan–Killiany and 18 subcortical gray matter regions (aparc+aseg output file^63,64^). The 268-region Shen atlas (Shen268 or sh268) is an MNI-space cortical and subcortical volumetric atlas based on resting-state fMRI clustering^65,66^. The 439-region CocoHCP439 atlas (Coco439 or co439) combines 358 cortical regions from the HCP multimodal parcellation^67^, defined using anatomical and functional connectivity gradients, with 12 subcortical regions from FreeSurfer aseg (further modified by FSL FIRST^68^; FSL 6.0), 30 thalamic nuclei derived from FreeSurfer 7.2.0 (ref. ^69^; 50 original outputs included many small nuclei, which were merged into the final set of 30), 27 cerebellar regions from the SUIT atlas^70^, and 12 additional subcortical nuclei from AAL3 (ref. ^71^). The FS86 and Coco439 atlases were defined based on each individual subject’s FreeSurfer surface and subcortical parcellations, whereas the Shen268 atlas was applied directly to MNI-resampled data for each subject. The FS86, Shen268 and Coco439 parcellations result in 3,655, 35,778 and 96,141 pairwise connectivity estimates, respectively.

### Measuring connectome prediction accuracy

A key consideration when assessing the performance of a connectome prediction model is the high degree of similarity of measured connectomes with the population mean. The Pearson correlation between connectomes of unrelated individuals can exceed 0.9, depending on the flavor, and a model that simply predicts the population mean for every subject can appear to have a very high prediction accuracy if it uses Pearson correlation as its metric (Fig. 2d, ‘pop. mean’ in bottom subpanel). One simple solution to this issue is to subtract the population mean calculated from training subjects before correlating measured and predicted connectomes; here, we refer to this metric as avgcorr_demean_ (equation (2)). However, this metric still cannot determine whether predictions faithfully capture individual variation, particularly when there is clustering within the population (Extended Data Fig. 1a).

Our goal here is to create individualized connectome predictions to understand the sources and consequences of across-subject variability. Thus, we also consider the identifiability of our predictions: whether our measured connectome for a subject is closer to the predicted connectome for that subject than the predicted connectome of any other subject. The top-1 accuracy (top1acc, equation (3)) is a winner-take-all measure that reflects the fraction of subjects for whom this is true. Specifically, it measures the fraction of subjects whose predicted connectome is more similar (higher Pearson correlation) to their measured connectome than to any other subject’s measured connectome (random chance 1/*n*_subj_ = 0.005 for 196 HCP-YA test subjects). For a more continuous measure of identifiability, more suitable to assessing imperfect predictions, we use an ‘average rank percentile’ measure (avgrank, equation (4)), which is the fractional rank of the similarity (Pearson correlation) of a subject’s measured and predicted connectomes compared with all other predicted connectomes, averaged across subjects (random chance, 0.5). Taken together, prediction accuracy and identifiability, as measured with avgcorr_demean_ and avgrank, convey a more complete picture of how well a model explains individuals’ connectomes.

The following metrics were used to compare predicted connectomes (denoted ${\hat{X}}_{s}$ for subject *s*) with measured connectomes (denoted *X*_*s*_) when evaluating model performance:

1 $${\rm{avgcorr}}=\frac{1}{{n}_{{\rm{subj}}}}\mathop{\sum }\limits_{s=1}^{{n}_{{\rm{subj}}}}{\rm{corr}}({X}_{s},{\hat{X}}_{s})$$

2 $$\begin{array}{l}{{\rm{avgcorr}}}_{{\rm{demean}}}=\displaystyle\frac{1}{{n}_{{\rm{subj}}}}\mathop{\sum }\limits_{s=1}^{{n}_{{\rm{subj}}}}{\rm{corr}}({X}_{s}-{\mu }_{{\rm{tr}}},{\hat{X}}_{s}-{\mu }_{{\rm{tr}}}),\,\\\quad\quad\quad\quad\text{where}\,\,{\mu }_{{\rm{tr}}}=\left(\displaystyle\frac{1}{{n}_{{\rm{subj}}}}\mathop{\sum }\limits_{s=1}^{{n}_{{\rm{subj}}}}{X}_{s}\right)\,\text{for training subjects}\,\end{array}$$

3 $$\begin{array}{l}{\rm{top1acc}}=\displaystyle\frac{1}{{n}_{{\rm{subj}}}}\mathop{\sum }\limits_{s=1}^{{n}_{{\rm{subj}}}}\left[{{\rm{argmax}}}_{a\in 1\ldots {n}_{{\rm{subj}}}}({\rm{corr}}({X}_{s},{\hat{X}}_{a}))=s\right],\,\\\quad\quad\quad\quad\text{where}\,\,[\cdot ]\,\text{represents an indicator function}\,\end{array}$$

4 $${\rm{avgrank}}=\frac{1}{{n}_{{\rm{subj}}}}\mathop{\sum }\limits_{s=1}^{{n}_{{\rm{subj}}}}\left(\frac{1}{{n}_{{\rm{subj}}}}\mathop{\sum }\limits_{a\ne s}^{{n}_{{\rm{subj}}}}[{\rm{corr}}({X}_{s},{\hat{X}}_{a}) < {\rm{corr}}({X}_{s},{\hat{X}}_{s})]\right)$$

In addition to these measures of prediction accuracy for the connectivity matrix values, we compute several common graph theoretical metrics to assess how faithfully the predicted connectomes capture the network properties of the measured connectomes. We use the Brain Connectivity Toolbox^72^ to compute average node strength, average betweenness centrality, characteristic path length, and modularity (Louvain community detection) for each measured and predicted connectome, and compute the Spearman rank correlation between the predicted and measured values for all test subjects for each metric.

### Model training

**Extended Data Fig. 8:**
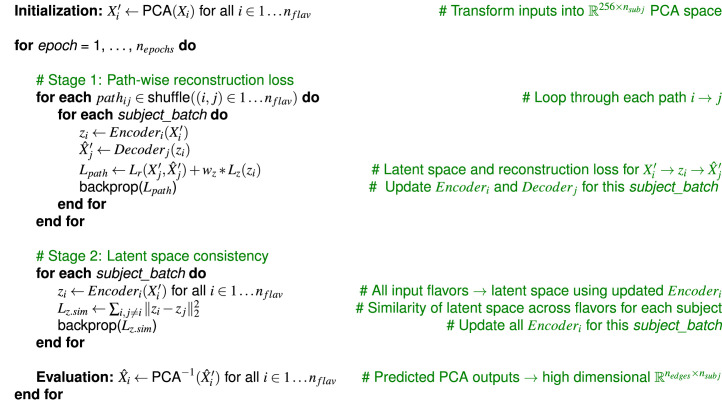
Krakencoder model training algorithm. Each model training epoch comprises two stages: path-wise reconstruction and inter-subject dispersion, and latent space consistency. See Extended Data Table 1 for loss function details (Extended Data Table 1).

Each model training epoch consists of two stages: (1) path-wise reconstruction and inter-subject dispersion and (2) latent space consistency. For a given path *i* → *j*, we loop through batches of subjects (*n*_batch_ = 41), apply Encoder_*i*_ to transform input flavor ${X}_{i}^{{\prime} }$ (256 × *n*_batch_) to latent representation *z*_*i*_ (128 × *n*_batch_), and Decoder_*j*_ to transform *z*_*i*_ to predicted output flavor ${\hat{X}}_{j}^{{\prime} }$ (256 × *n*_batch_). Path-wise reconstruction loss and inter-subject dispersion terms (*L*_r.corrI_, *L*_r.mse_, *L*_r.marg_) are computed per batch from ${X}_{j}^{{\prime} }$ and ${\hat{X}}_{j}^{{\prime} }$. These reconstruction losses collectively penalize reconstruction error in both absolute MSE and correlation terms, and promote inter-subject separation in the reconstruction space. Path-wise latent space dispersion loss (*L*_z.corrI_, *L*_z.dist_), computed from *z*_*i*_, promotes inter-subject separation in the latent space. This forward pass and backpropagation is repeated for each batch of training subjects, before moving on to the next training path. The order in which paths are selected is randomly reshuffled each epoch. The second stage of each training epoch, latent space consistency, enforces similarity of the latent space representations from each input flavor. We re-compute *z*_*i*_ for all input flavors from the encoders updated in the path-wise stage, and then compute *L*_z.sim_ as the total MSE between the set of *z*_*i*_ matrices for all flavors. Explicit weights were applied to the reconstruction MSE loss term *L*_r.mse_ (*w*_rm_ = 1,000), the combined path-wise latent space dispersion loss *L*_*z*_ (*w*_*z*_ = 10), and the latent space intra-subject consistency loss *L*_z.sim_ (*w*_zs_ = 10,000). See Extended Data Fig. 8 for pseudocode of training procedure, and Extended Data Table 1 for details of loss function terms.

After each training epoch, we compute predicted connectomes for all paths on a held-out set of 80 validation subjects, and the inverse PCA transform (precomputed on the training data only) is applied to compute ${\hat{X}}_{j}\in {{\mathbb{R}}}^{{n}_{{\rm{edges}}}}$ from ${\hat{X}}_{j}^{{\prime} }\in {{\mathbb{R}}}^{256}$. Reconstruction accuracy and identifiability are computed in this high-dimensional data space. Loss term weights were tuned empirically to balance the reconstruction (avgcorr_demean_) and identifiability (avgrank) performance metrics in the validation dataset. Extended Data Fig. 1b illustrates this trade-off when tuning MSE reconstruction loss.

The model was implemented in PyTorch 1.10, using the AdamW optimizer with learning rate 10^−4^. Model weights were regularized by weight decay 10^−2^, as well as dropout in all encoders and decoders (dropout rate 0.5). The model was trained for 5,000 epochs, which took approximately 36 h on an Nvidia A100 GPU. Due to the regularization and the largely linear nature of the model, we did not observe pronounced overfitting. Validation performance plateaued after 500–1,000 epochs, and we selected the 2,000-epoch checkpoint for further evaluation.

### Comparing family structure and predicting demographics

To examine how the connectomes (predicted and measured) reflect family structure, we first compute the averaged latent vector in the Krakencoder for each subject from either all 15 flavors (fusion) or the nine FC (fusionFC) and six SC (fusionSC) separately, and then compute an *n*_subj_ × *n*_subj_ inter-subject latent similarity matrix using Pearson correlation. These pairwise similarities are grouped according to the inter-subject relationship. For comparison, we also compute the inter-subject similarity for all 15 flavors of the high-dimensional measured connectome data, average these 9, 6 or 15 *n*_subj_ × *n*_subj_ similarity matrices, and group them according to relatedness. Due to the high baseline similarity in observed data, latent and observed data similarity measures were independently z-scored by the mean and standard deviation of the similarity of unrelated subjects for visual comparison. Differences of receiver operating characteristic (ROC) separability of the distributions (for example, separability of DZ and MZ distributions in latent space, compared with separability of DZ and MZ in observed data) was assessed through permutation testing (10,000 matched permutations).

For demographic predictions, we used a linear support vector classifier to predict sex, and kernel ridge regression with linear kernel to predict age and cognition^73^. For the Krakencoder, models were fitted using cross-validation on the average latent vectors for the 683 subjects used to train the Krakencoder model, and tested on the 196 held-out subjects. For the observed connectome data, we computed the inter-subject cosine similarity matrix of each connectome flavor, and used the averaged similarity matrices as input into the model, using the same training and testing splits as for the Krakencoder. Regression hyperparameters were selected by nested cross-validation grid search. Variability of prediction accuracy was assessed through bootstrap resampling (*n* = 100), and significant differences between Krakencoder and observed data models was assessed using two-sided permutation testing of matched bootstrap samples (10,000 permutations).

### Spatial variation in edge prediction accuracy

We assessed the spatial variability of prediction accuracy to determine whether certain brain regions are more accurately predicted than others. For fusion-parc, fusionFC-parc and fusionSC-parc (fusion predictions made without the parcellation being predicted), the prediction accuracy for each edge is the Pearson correlation between the predicted and measured values for the 196 held-out test subjects. To simplify visualization and enable comparison across varied size parcellations, each region is assigned to one of seven cortical resting-state networks^74^ or subcortical and cerebellar networks. The edge-wise accuracies (region × region) are then grouped and averaged into a 9 × 9 network representation for each connectome flavor. In the top block of Extended Data Fig. 2a, the network-averaged edge prediction accuracy is presented for each of the fusion types as input (rows) and each connectivity flavor as output (columns). The bottom block shows the baseline network-averaged edge similarity comparing measured connectomes of unrelated age- and sex-matched subjects and related individuals. The figure demonstrates that the Krakencoder’s prediction accuracy is relatively uniform throughout the cortex and subcortex. Networks with lower Krakencoder predictability (for example, the limbic network), also show low self-retest measurement reliability (bottom row). In Extended Data Fig. 2b, each network × network heatmap in Extended Data Fig. 2a is correlated with the corresponding self-retest measurement reliability (bottom row), to quantify the consistency of the spatial pattern of edge-wise accuracies with the reliability of the edge-wise measurements. We see that the spatial pattern of the edge-wise accuracy of the Krakencoder’s fused intra- and inter-modality predictions is as similar to the spatial pattern of the edge-wise self-retest measurement reliability as the MZ twins’ spatial patterns of edge-wise reliability. Extended Data Fig. 3 summarizes the edge-wise prediction accuracy at a region level for each connectivity flavor in its own parcellation, showing a similar agreement between prediction and measurement reliability.

To quantify the relationship between SC–FC predictions and cortical hierarchy, we first correlate regional prediction accuracy with an estimate of the cortical hierarchy gradient derived from functional connectivity^75^. We find a significant positive correlation (Spearman *r* = 0.249, *P*_spin_ = 0.019, 10,000 spin-test rotations^76^). Conversely, if we correlate corresponding rows in the square measured and predicted connectome matrices, we find that predicted connections to high-level cortex are less coupled to their measured rows than low-level cortex (Spearman *r* = −0.191, *P*_spin_ = 0.116; Extended Data Fig. 4b).

### Network-level sensitivity analyses

Regions were grouped into seven cortical resting-state networks^74^ or subcortical and cerebellar networks. To perform the sensitivity analyses, every connectome edge was set to the population mean of the training set, except for the columns and rows corresponding to the regions within the network of interest, which were left untouched (Fig. 4a). The fusion latent space representations (fusion, fusionSC and fusionFC) were re-calculated for each individual and used to predict, first, each connectome flavor, and second, age, sex and total cognition using re-fitted kernel ridge regression models.

### Training comparable models from literature

The Krakencoder model incorporates six flavors of SC (two per parcellation) and nine flavors of FC (three per parcellation). These two previous SC–FC prediction frameworks are designed to predict a single flavor of FC from SC. Thus, we trained 12 separate models for each of the two frameworks for all combinations of SC (SC_dt_ or SC_pr_) and FC (FC or FC_pcorr_). FC_gsr_ was excluded due to computational constraints and relative redundancy with FC.

A note on computational efficiency: training all 12 deepnet models required approximately 140 GPU hours on an Nvidia A100 (4 × 5 h for FS86, 4 × 10 h for Shen268, and 4 × 20 h for Coco439), and all 12 graphnet models required 1,200 GPU hours (4 × 50 h for FS86, 4 × 100 h for Shen268, 4 × 150 h for Coco439). For comparison, the Krakencoder required only 30 GPU hours to train a model for all 15 × 15 = 225 prediction paths simultaneously. When applying the Krakencoder model to new connectome data, the computational requirements are minimal and can even be done using free cloud-based platforms such as Google Colab.

### Reporting summary

Further information on research design is available in the Nature Portfolio Reporting Summary linked to this article.

## Online content

Any methods, additional references, Nature Portfolio reporting summaries, source data, extended data, supplementary information, acknowledgements, peer review information; details of author contributions and competing interests; and statements of data and code availability are available at 10.1038/s41592-025-02706-2.

## Supplementary information

Supplementary Information Supplementary Figs. 1 and 2.

Reporting Summary

## Figures and Tables

**Extended Data Table 1:**
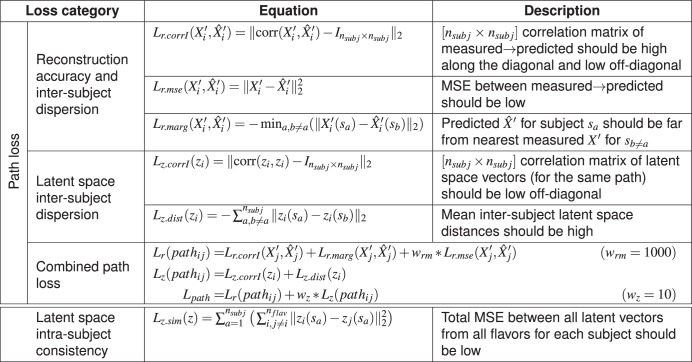
Loss function summary

**Extended Data Table 2:**
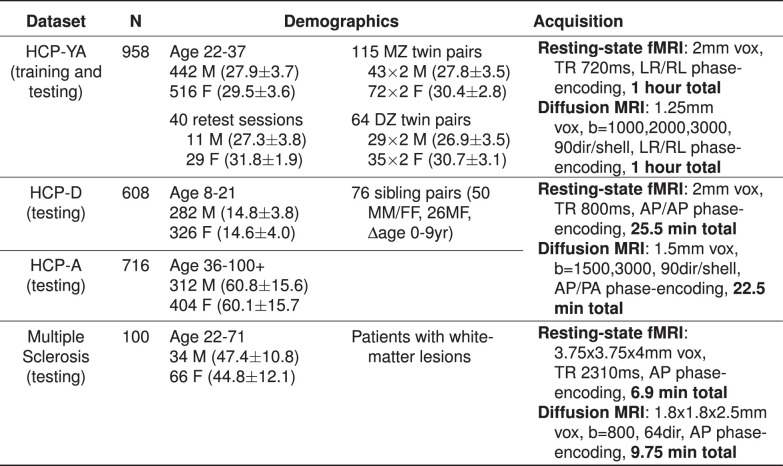
Summary of datasets used in this study

## Data Availability

Preprocessed data for this study are available for download from the Human Connectome Project (www.humanconnectome.org). Users must agree to data use terms for the HCP before being allowed access to the data and ConnectomeDB; details are provided at https://www.humanconnectome.org/study/hcp-young-adult/data-use-terms. Data from the HCP Aging and HCP Development studies can be downloaded as part of the HCP-Lifespan 2.0 release, distributed by the NIMH Data Archive (https://nda.nih.gov). See https://www.humanconnectome.org/study/hcp-lifespan-aging/data-releasesand https://www.humanconnectome.org/study/hcp-lifespan-development/data-releasesfor more information about data use terms. Post-processed connectomes and related input files can be made available upon reasonable request from the corresponding author K.W.J., subject to HCP data use restrictions described above.

## Extended data

are available for this paper at 10.1038/s41592-025-02706-2.

## Code availability

Code is available at https://github.com/kjamison/krakencoder. Post-processing code for functional connectivity can be found here: https://github.com/kjamison/fmriclean. Source code is provided under the MIT License (https://opensource.org/license/mit). Additional data files needed for running the code are available at https://osf.io/dfp92.
