# Supplementary material for: Krakencoder: a unified brain connectome translation and fusion tool
Source: Nat Methods. 2025 Jun 5;22(7):1583–92. doi: 10.1038/s41592-025-02706-2 (PMC12240824; doi:10.1038/s41592-025-02706-2)
Supplement: Supplementary file 1 — Supplementary Figs. 1 and 2. [file 41592_2025_2706_MOESM1_ESM.pdf]

nature methods

Supplementary information https://doi.org/10.1038/s41592-025-02706-2

Krakencoder: a unified brain connectome translation and fusion tool

In the format provided by the authors and unedited

Supplemental Information for
Krakencoder: A unified connectome translation and fusion tool

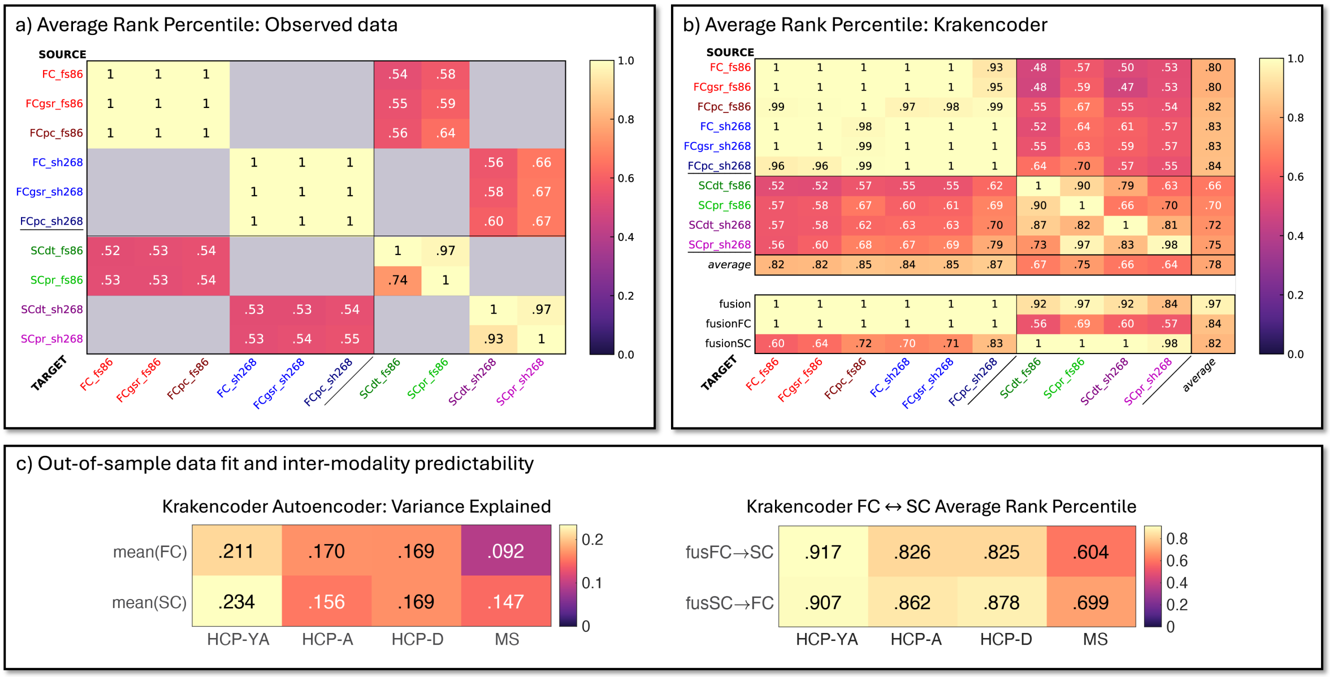

Supplementary Figure 1. Krakencoder performance in individuals with multiple sclerosis (out-of-sample and out-of-distribution test set) Average rank percentiles of identifiability in 100 individuals with multiple sclerosis using (**a**) the observed SC and FC (within atlas comparisons only) and (**b**) the Krakencoder's individual arm predictions (top) and the fusion/fusionSC/fusionFC predictions (bottom). **c.** For each study, data for flavor X_i is encoded and then decoded, and variance explained $(1 - var(X_i - \hat{X}_i)/var(X_i))$ is averaged across FC and SC flavors. This heatmap roughly summarizes how well the FC and SC data from each study are explained by the Krakencoder latent space.

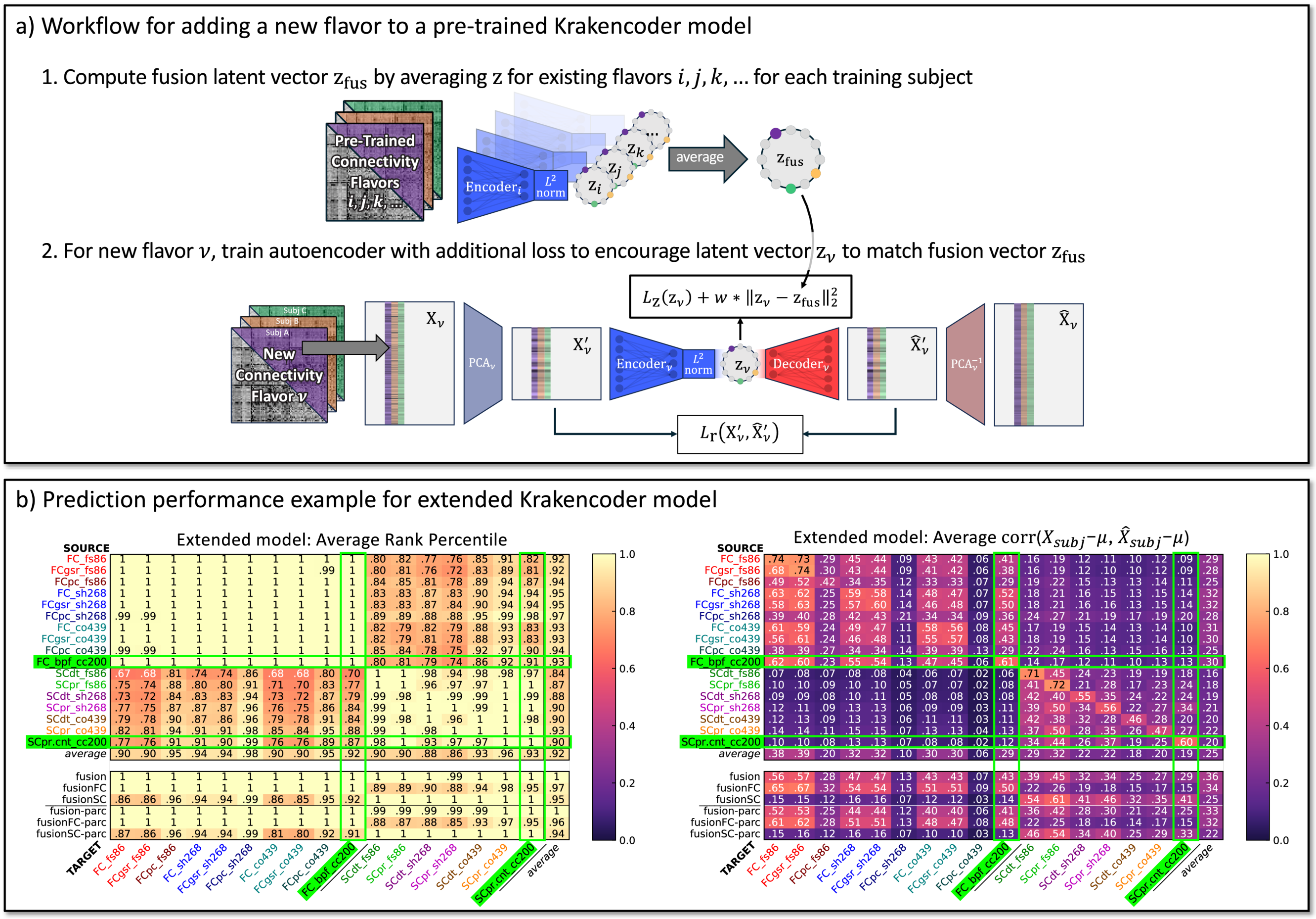

Supplementary Figure 2. A pre-trained Krakencoder model can be extended to new flavors a. To add a new flavor, we first compute the fusion representation $z_{\text{fus}} \in \mathbb{R}^{128 \times n_{subj}}$ by averaging the latent vector for each flavor in the existing model. With new connectome data $X_v \in \mathbb{R}^{n_{edges} \times n_{subj}}$, we compute new PCA transformations and train a new autoencoder, using the same reconstruction and latent loss terms L_r and L_z as the original training, but with an additional loss term to force the new latent z_v to be similar to z_{fus}. **b.** Connectome prediction performance after adding two new flavors to the original 15 flavor model. New flavors shown here use a previously unseen parcellation (cc200*), bandpass-filtered time series for FC (original flavors use a high-pass filter), and un-normalized streamline count for SC (original flavors use pairwise volume normalization). Prediction identifiability (avgrank, left) and reconstruction accuracy ($\text{avgcorr}_{\text{demean}}$, right) for these new flavors are highlighted in green. (* Craddock, R. C., James, G. A., Holtzheimer, P. E., Hu, X. P. Mayberg, H. S. A whole brain fMRI atlas generated via spatially constrained spectral clustering. *Human Brain Mapping*. 33, 1914–1928 [2012])
