# Supplementary material for: Krakencoder: a unified brain connectome translation and fusion tool
Source: Nat Methods. 2025 Jun 5;22(7):1583–92. doi: 10.1038/s41592-025-02706-2 (PMC12240824; doi:10.1038/s41592-025-02706-2)
Supplement: Supplementary file 2 — Reporting Summary [file 41592_2025_2706_MOESM2_ESM.pdf]

nature portfolio

Corresponding author(s): Keith W Jamison

Last updated by author(s): 12/12/2024

Reporting Summary

Nature Portfolio wishes to improve the reproducibility of the work that we publish. This form provides structure for consistency and transparency in reporting. For further information on Nature Portfolio policies, see our Editorial Policies and the Editorial Policy Checklist.

Statistics

For all statistical analyses, confirm that the following items are present in the figure legend, table legend, main text, or Methods section.

n/a	Confirmed	
☐	☒	The exact sample size (*n*) for each experimental group/condition, given as a discrete number and unit of measurement
☒	☐	A statement on whether measurements were taken from distinct samples or whether the same sample was measured repeatedly
☐	☒	The statistical test(s) used AND whether they are one- or two-sided<br>*Only common tests should be described solely by name; describe more complex techniques in the Methods section.*
☒	☐	A description of all covariates tested
☐	☒	A description of any assumptions or corrections, such as tests of normality and adjustment for multiple comparisons
☐	☒	A full description of the statistical parameters including central tendency (e.g. means) or other basic estimates (e.g. regression coefficient) AND variation (e.g. standard deviation) or associated estimates of uncertainty (e.g. confidence intervals)
☐	☒	For null hypothesis testing, the test statistic (e.g. *F*, *t*, *r*) with confidence intervals, effect sizes, degrees of freedom and *P* value noted<br>*Give P values as exact values whenever suitable.*
☒	☐	For Bayesian analysis, information on the choice of priors and Markov chain Monte Carlo settings
☒	☐	For hierarchical and complex designs, identification of the appropriate level for tests and full reporting of outcomes
☐	☒	Estimates of effect sizes (e.g. Cohen's *d*, Pearson's *r*), indicating how they were calculated

Our web collection on statistics for biologists contains articles on many of the points above.

Software and code

Policy information about availability of computer code

Data collection	No software was used for data collection.
Data analysis	Python code for this study is available at https://github.com/kjamison/krakencoder (Python >= 3.8, pytorch 1.13.1, see repository for detailed requirements). Post-processing code for functional connectivity (FC) can be found here: https://github.com/kjamison/fmriclean (Python >= 3.8, Nilearn>=0.7.0). Other software included FSL 6.0, MRtrix3 (3.0_RC3), FreeSurfer 7.2.0 (for thalamic parcellation), Brain Connectivity Toolbox (bctpy 0.6.1)

For manuscripts utilizing custom algorithms or software that are central to the research but not yet described in published literature, software must be made available to editors and reviewers. We strongly encourage code deposition in a community repository (e.g. GitHub). See the Nature Portfolio guidelines for submitting code & software for further information.

Data

Policy information about availability of data

All manuscripts must include a data availability statement. This statement should provide the following information, where applicable:

- Accession codes, unique identifiers, or web links for publicly available datasets
- A description of any restrictions on data availability
- For clinical datasets or third party data, please ensure that the statement adheres to our policy

Preprocessed data for this study are available for download from the Human Connectome Project (www. humanconnectome.org). Users must agree to data use

terms for the HCP before being allowed access to the data and ConnectomeDB, details are provided at https://www.humanconnectome.org/study/hcp- young-adult/data-use-terms. Data from the HCP Aging and HCP Development studies can be downloaded as part of the HCP-Lifespan 2.0 release, distributed by the NIMH Data Archive (https://nda.nih.gov). See https://www.humanconnectome.org/study/hcp-lifespan-aging/data-releases and https: //www.humanconnectome.org/study/hcp-lifespan-development/data-releases for more information about data use terms.

Additional data files for applying models from this study are available at https:// osf.io/dfp92.

Post-processed connectomes and related input files can be made available upon reasonable request from corresponding author K.J., subject to HCP data use restrictions described above.

Human research participants

Policy information about studies involving human research participants and Sex and Gender in Research.

Reporting on sex and gender	We use the self-reported biological sex provided by the Human Connectome Project.
Population characteristics	For HCP-YA, subjects were 958 healthy young adults aged 22-37 (442 males with mean age 27.9, 516 females with mean age 29.5), including 115 monozygotic twin pairs, (42 male pairs, 72 female pairs), 64 dizygotic twin pairs (29 male pairs, 35 female pairs), and<br><br>For HCP-Development, subjects were 608 healthy children and adolescents aged 8-21 (282 males with mean age 14.8, 326 females with mean age 14.6), including 76 sibling pairs.<br><br>For HCP-Aging, subjects were 716 healthy older adults aged 36-100 (312 males with mean age 60.8, 404 females with mean age 60.1).<br><br>For Multiple Sclerosis data, subjects were 100 adults aged 22-71 (34 males with mean age 47.4, 66 females with mean age 44.8)
Recruitment	We did not recruit subjects for this study.
Ethics oversight	This research was approved by the Weill Cornell Medicine Institutional Review Board for an exemption on deidentified public data

Note that full information on the approval of the study protocol must also be provided in the manuscript.

Field-specific reporting

Please select the one below that is the best fit for your research. If you are not sure, read the appropriate sections before making your selection.

☒ Life sciences ☐ Behavioural & social sciences ☐ Ecological, evolutionary & environmental sciences

For a reference copy of the document with all sections, see nature.com/documents/nr-reporting-summary-flat.pdf

Life sciences study design

All studies must disclose on these points even when the disclosure is negative.

Sample size	We did not collect the data, so no sample size calculation was performed. We used all available data for each study, and demonstrate significant effects with this size.
Data exclusions	Subjects were excluded if they did not complete all resting-state or diffusion MRI scans, or if they had noted problems during acquisition or preprocessing, leaving 958 for HCP-YA, 608 for HCP-Development, and 716 for HCP-Aging.
Replication	We used HCP-Development and HCP-Aging datasets to test the generalizability on our model that was trained on HCP-YA. HCP Lifespan data differs from HCP-YA in both age range and acquisition parameters, with Lifespan acquiring less than half as much fMRI and dMRI data as HCP-YA. HCP Lifespan's more conservative acquisition parameters and scan durations more closely match those in many modern neuroimaging research studies. HCP Lifespan studies were also collected across four different sites using commercially available 3T MRI scanners while the HCP-YA data were all acquired on a customized MRI scanner at a single site. We also tested our tool on data from patients with multiple sclerosis, using a much shorter and lower quality acquisition. Our model's connectome predictions were above chance levels for all replication studies, though performance was lower than the initial high-quality HCP data.
Randomization	When randomizing subjects for training and testing, or for cross-validation, we made sure related subjects were kept in the same split, to avoid data leakage.
Blinding	Blinding was not relevant for our study, as there were no treatment groups.

Reporting for specific materials, systems and methods

We require information from authors about some types of materials, experimental systems and methods used in many studies. Here, indicate whether each material, system or method listed is relevant to your study. If you are not sure if a list item applies to your research, read the appropriate section before selecting a response.

Materials & experimental systems

n/a	Involved in the study
☒	☐ Antibodies
☒	☐ Eukaryotic cell lines
☒	☐ Palaeontology and archaeology
☒	☐ Animals and other organisms
☒	☐ Clinical data
☒	☐ Dual use research of concern

Methods

n/a	Involved in the study
☒	☐ ChIP-seq
☒	☐ Flow cytometry
☐	☒ MRI-based neuroimaging

Magnetic resonance imaging

Experimental design

Design type

Resting-state functional fMRI

Design specifications

For HCP-Young Adult, two 15 minute rsfMRI scans per session, with 2 sessions over consecutive days. 40 subjects underwent a complete re-scan 1-11 months later, to examine test-retest reliability.

For HCP-Aging and HCP-Development, two scans with length 6:41 per session, with 2 sessions over consecutive days.

Behavioral performance measures

No behavioral measures were taken during the resting state scans. Subjects were instructed to stay awake and fixate on a central cross-hair.

Acquisition

Imaging type(s)

Resting-state functional MRI, Diffusion MRI

Field strength

3T

Sequence & imaging parameters

HCP-YA Functional MRI: Gradient-echo EPI. 2.0mm isotropic voxels, TR/TE=720/33.1ms, 8x multi-band acceleration, FOV 208x180mm, FA=52 deg, 72 slices. Two sessions included a pair of 15 minute scans, with R>>L and L>>R phase-encoding, for a total of 60 minutes. Each scan had a pair of R>>L and L>>R Spin-echo B0 fieldmaps for susceptibility correction. Customized Siemens 'ConnectomeS' scanner with 100mT/m gradient coil.

HCP-YA Diffusion MRI: Spin-echo EPI, 1.25mm isotropic voxels, TR/TE=5520/89.5ms, 3x multi-band acceleration, FOV 210x180mm, FA/refocus=78/160 deg, 111 slices. b=1000,2000,3000 s/mm^2 with 90 directions/shell and b=0 every 16 volumes. Session includes 6 10 minute scans, with all directions repeated for R>>L and L>>R phase-encoding, for a total of 60 minutes. Customized Siemens 'ConnectomeS' scanner with 100mT/m gradient coil.

HCP-YA Anatomical MRI: T1-weighted were 3D-MPRAGE with 0.7mm isotropic voxels (FOV=224x224mm, matrix=320, 256 sagittal slices, TR/TE/TI=2400/2.14/1000ms, FA=8 deg, GRAPPA=2). T2-weighted scans were acquired using a variable flip angle turbo spin-echo sequence (Siemens SPACE) with 0.7mm isotropic voxels (FOV matched MPRAGE, TR/TE=3200/565ms, GRAPPA=2). Customized Siemens 'ConnectomeS' scanner with 100mT/m gradient coil.

HCP-Lifespan Functional MRI: Gradient-echo EPI. 2.0mm isotropic voxels, TR/TE=800/37ms, 8x multi-band acceleration, FOV 208x180mm, FA=52 deg, 72 slices. Two sessions included a pair of 6:37 scans, with A>>P and P>>A phase-encoding, for a total of 25.5 minutes. Each scan had a pair of A>>P and P>>A spin-echo B0 fieldmaps for susceptibility correction. Siemens Prisma scanner with 80 mT/m gradient coil.

HCP-Lifespan Diffusion MRI: Spin-echo EPI, 1.5mm isotropic voxels, TR/TE=3220/89.2ms, 4x multi-band acceleration, FOV=210x210mm, FA/refocus=78/160 deg. 92 slices. b=1500, 3000 s/mm^2 with 92 directions/shell and b=0 every 16 volumes. Session includes 4 5:37 scans, with all directions repeated for A>>P and P>>A phase-encoding, for a total of 22.5 minutes.

HCP-Lifespan Anatomical MRI: T1-weighted were multi-echo 3D-MPRAGE with 0.8mm isotropic voxels (FOV=256x240mm, matrix=320, 208 sagittal slices, TR=2500ms, Multi-TE=1.8/3.6/5.4/7.2 ms, TI=1000ms, FA=8 deg, GRAPPA=2). T2-weighted scans were acquired using a variable flip angle turbo spin-echo sequence (Siemens SPACE) with 0.8mm isotropic voxlels (FOV matched MPRAGE, TR/TE=3200/564ms, GRAPPA=2). Siemens Prisma scanner with 80 mT/m gradient coil.

Multiple Sclerosis Functional MRI: Gradient-echo EPI. 3.75x3.75x4mm voxels, TR/TE=2310/30ms, FOV 240x240mm,

FA=70deg, 38 slices. Each subject has a single 6:56 scan, with A>>P phase encoding, and a GRE fieldmap.

Multiple Sclerosis Diffusion MRI: Spin-echo EPI, 1.8x1.8x2.5mm voxels, TR/TE=9000/95ms, FOV=230x230mm, FA/refocus=90/180 deg. 60 slices. b=800 s/mm^2 with 65 directions and a single b=0. Total duration 9:45 with A>>P phase-encoding.

Area of acquisition: Whole-brain FOV

Diffusion MRI: ☒ Used ☐ Not used

Parameters: HCP-YA 1.25mm, b=1000,2000,3000s/mm2 with 90 directions/shell, collected RL+LR. HCP-Lifespan 1.5mm, b=1500,3000s/mm2 with 92 directions/shell, collected AP+PA.

Diffusion MRI were preprocessed using topup+eddy, and coregistered to anatomy. Additional processing with MRtrix3 included bias correction, constrained spherical deconvolution (multi-shell, multi-tissue FOD estimation, lmax=8), and both deterministic (SD_STREAM) and probabilistic (iFOD2+anatomical constraint) whole-brain tractography with dynamic white-matter seeding. Regional connectivity was computed as the number of streamlines terminating in each ROI pair, divided by the total volume of those regions.

Preprocessing

Preprocessing software: We used data from the HCP-YA S1200 release and the HCP-Lifespan 2.0 release. For HCP-YA fMRI, dMRI, and anatomy, and for HCP-Lifespan fMRI and anatomy, we used minimally preprocessed data provided by the HCP consortium using the Minimal Processing Pipeline (Glasser 2013), which combines tools from FSL 5.0.9, FreeSurfer 5.3, and Connectome Workbench. HCP-Lifespan dMRI was preprocessed separately from the raw data using the same pipeline. Functional MRI data were further post-processed with a custom Python3 package using Nilearn tools (0.7.0) for additional denoising, volume censoring, ROI time series extraction, and FC extraction (https://github.com/kjamison/fmriclean). Diffusion MRI were further processed using MRtrix3 (3.0_RC3), including tractography and construction of SC matrices.

Normalization: Volumetric normalization was performed during the HCP Minimal Preprocessing Pipeline using FNIRT.

Normalization template: HCP fMRI data have been volume normalized to the MNI152v6asym template.

Noise and artifact removal: HCP fMRI data have been denoised using a very liberal high-pass filter (0.0005 Hz), motion parameter regression, and ICA-FIX (Salimi-Korshidi, 2014) to automatically reject components classified as structured noise based on spatial and temporal features. We performed additional denoising using techniques from Nilearn (scripts here: https://github.com/kjamison/fmriclean) to regress 5 eigenvectors each from CSF and WM tissue masks (aCompCor, Behzadi 2007), as well as 24 motion parameter time series (Power 2014). HCP-YA data was high-pass filtered > 0.01 Hz, and HCP-Lifespan data > 0.008 Hz.

Volume censoring: Outlier volumes were identified using motion and global signal (motion derivative > 0.9mm, or global signal >5σ). The first 10 volumes from each scan were also ignored.

Statistical modeling & inference

Model type and settings: We examined family structure within the data by computing inter-subject similarity matrices and grouping these pairwise similarities by familial relationship. ROC separability of the distributions (e.g., separability of DZ and MZ distributions of similarity predicted from model A, compared to separability of DZ and MZ similarity in model B) was assessed through permutation testing (10000 matched permutations).

For demographic predictions, we used a linear support vector classifier (SVC) to predict sex, and kernel ridge regression with linear kernel to predict age and cognition. Regression hyperparameters were selected by nested cross-validation grid search within the training set. Variability of prediction accuracy was assessed through bootstrap resampling (N=100), and significant differences between models was assessed using permutation testing of matched bootstrap samples (10000 permutations).

Effect(s) tested: No task or stimulus was tested, as this is a resting-state and diffusion MRI study.

Specify type of analysis: ☐ Whole brain ☒ ROI-based ☐ Both

Anatomical location(s): The 86-region FreeSurfer atlas combines 68 cortical gyri from Desikan-Killiany and 18 subcortical gray matter regions (aparc+aseg). The 268-region Shen atlas is an MNI-space cortical and subcortical volumetric atlas based on resting-state fMRI clustering (Shen 2013). The 439-region CocoHCP439 atlas combines 358 cortical regions from the HCP multimodal parcellation, with 12 subcortical regions from FreeSurfer "aseg" (further refined by FSL FIRST), 30 thalamic nuclei derived from FreeSurfer7 (50 original outputs included many small nuclei, which were merged into the final set of 30), 27 cerebellar regions from the SUIT atlas, and 12 additional subcortical nuclei from AAL3. The FS86 and CocoHCP439 atlases were defined based on each individual subject's FreeSurfer surface and subcortical parcellations, whereas the Shen268 atlas was applied directly to MNI-resampled data for each subject.

Statistic type for inference (See Eklund et al. 2016): We did not perform voxel-wise or cluster-wise analyses

Correction: We used Benjamini-Hochberg false discovery rate correction to report p-values across grouped comparisons, such as family group separability or demographic predictions.

Models & analysis

n/a	Involved in the study
☐	☒ Functional and/or effective connectivity
☐	☒ Graph analysis
☐	☒ Multivariate modeling or predictive analysis

Functional and/or effective connectivity

We use several measures of functional connectivity between ROIs: Pearson correlation (FC), Pearson correlation following global signal regression (FCgsr), and Tikhonov-regularized partial correlation (FCpcorr). For Tikhonov-regularization, the regularization parameter was selected that maximized the similarity of the regularized precision matrix and the group averaged unregularized precision matrix. The specific parameter value ranged from 0.06 to 0.57, depending on the parcellation (FS86, Shen268, CocoHCP439) and dataset (HCP-YA, HCP-Development, HCP-Aging).

Graph analysis

We report mean node strength (sum of the absolute value of edges for each node, averaged across nodes), mean betweenness centrality (weighted, averaged across nodes), characteristic path length, and modularity (Louvain community detection, weighted and undirected). Graphs were not thresholded or binarized before computing graph metrics. All graph metrics were computed using the Brain Connectivity Toolbox.

Multivariate modeling and predictive analysis

We examined family structure within the data by computing inter-subject similarity matrices and grouping these pairwise similarities by familial relationship. ROC separability of the distributions (e.g., separability of DZ and MZ distributions of similarity predicted from model A, compared to separability of DZ and MZ similarity in model B) was assessed through permutation testing (10000 matched permutations).

For demographic predictions, we used a linear support vector classifier (SVC) to predict sex, and kernel ridge regression with linear kernel to predict age and cognition. Regression hyperparameters were selected by nested cross-validation grid search within the training set. Variability of prediction accuracy was assessed through bootstrap resampling (N=100), and significant differences between models was assessed using permutation testing of matched bootstrap samples (10000 permutations).
